# Pedestrian exposure to black carbon and PM_2.5_ emissions in urban hot spots: new findings using mobile measurement techniques and flexible Bayesian regression models

**DOI:** 10.1038/s41370-021-00379-5

**Published:** 2021-08-28

**Authors:** Honey Dawn Alas, Almond Stöcker, Nikolaus Umlauf, Oshada Senaweera, Sascha Pfeifer, Sonja Greven, Alfred Wiedensohler

**Affiliations:** 1 Leibniz Institute for Tropospheric Research (TROPOS), Leipzig, Germany; 2 Humboldt-Universität zu Berlin, Berlin, Germany; 3 Ludwig-Maximilians-Universität München (LMU), Munich, Germany; 4 Universität Innsbruck, Innsbruck, Austria

**Keywords:** Air Pollution, Criteria Pollutants, Environmental Monitoring, Personal Exposure, Particulate Matter, New Approach Methodologies (NAMs)

## Abstract

**Background:**

Data from extensive mobile measurements (MM) of air pollutants provide spatially resolved information on pedestrians’ exposure to particulate matter (black carbon (BC) and PM_2.5_ mass concentrations).

**Objective:**

We present a distributional regression model in a Bayesian framework that estimates the effects of spatiotemporal factors on the pollutant concentrations influencing pedestrian exposure.

**Methods:**

We modeled the mean and variance of the pollutant concentrations obtained from MM in two cities and extended commonly used lognormal models with a lognormal-normal convolution (logNNC) extension for BC to account for instrument measurement error.

**Results:**

The logNNC extension significantly improved the BC model. From these model results, we found local sources and, hence, local mitigation efforts to improve air quality, have more impact on the ambient levels of BC mass concentrations than on the regulated PM_2.5_.

**Significance:**

Firstly, this model (logNNC in bamlss package available in R) could be used for the statistical analysis of MM data from various study areas and pollutants with the potential for predicting pollutant concentrations in urban areas. Secondly, with respect to pedestrian exposure, it is crucial for BC mass concentration to be monitored and regulated in areas dominated by traffic-related air pollution.

## Introduction

Adverse health effects of traffic-related air pollutants (TRAPs) .have motivated the creation of mitigation policies across cities in Europe [1] which are evaluated through the continuous monitoring of the criteria pollutants in fixed locations. However, exposure to unregulated TRAPs such as black carbon (BC), which has more impact on health than PM_2.5_ mass concentration (PM with aerodynamic diameter <2.5 micrometer (µm)) [2] and is highly variable in space, may not be fully captured by fixed stations [3–5]. Nowadays, mobile measurements (MM) are done to determine the spatial distributions of TRAPs. The high spatial resolution data it provides have allowed the identification of hot spots and their sources [3, 6], and investigation of pollutants in urban areas where placing monitoring stations proves challenging. This spatially resolved data are particularly beneficial for analyzing the exposure of pedestrians to pollutants and for creating mitigation strategies to address air pollution.

In MM studies, inference is often based on descriptive summary statistics indicating the association of pollutant concentration with single environmental factors individually [7–9]. Thereby, pollutant measurements are typically spatially and/or temporally aggregated and spatial context (like street type) often serves as surrogate for traffic situation [3, 10–15]. This approach is recommended [11, 14, 16, 17] for determining the typical spatial distribution of pollutant concentration under a variety and combinations of environmental and meteorological conditions. Few studies have quantified the effects of several predictor variables on the spatial variability of the target pollutants. Riley et al. [18] used principal component analysis on data obtained from MM aboard a vehicle driving through neighborhoods close to an interstate. Rivas et al. [19] (and references therein) focused on commuters’ exposure in various transportation modes using regression models with multiple predictor variables to quantify the contributions of different determinants to exposure. The study closest to our objective is that of Yu et al. [20], which used a regression model on collocated MM performed along parallel streets to quantify contributing factors.

More generally, related statistical approaches to modeling air pollution based on the spatiotemporal context are often referred to as “land use regression” (LUR) [21], which has been applied to MM of BC [22–27] and other pollutants [28–30]. Applied procedures comprise multiple (log-)linear regression with different variable selection techniques and possibly extended to generalized additive models (GAM), but also machine learning techniques [23, 31]. LUR often focuses on predictive modeling [21, 31], but also on interpretation of model coefficients [29, 30]. While various model alternatives are considered, limited attention is given to probability distributions implied by the selected model. The least squares fit, conducted i.a. in linear regression, assumes normally (or Gaussian) distributed concentrations where all observations are assumed to have constant variance (homoscedasticity) and to be uncorrelated. When modeling log-concentrations [20, 29, 32–34], this translates to a lognormal distribution assumption for the original concentrations. The methodological novelties presented in this paper can be understood as extensions of this approach in the context of a distributional perspective.

The most important methodological contribution is that we explicitly address measurement error arising from normally distributed noise in BC measurements by proposing a lognormal-normal convolution (logNNC) [35] model. Instrument measurement error has been deemed an important issue in recent statistical approaches for air pollution-related epidemiological studies [33, 36, 37]. Although it averages to zero, i.e., it is not systematic itself, neglecting the error still leads to biased coefficient estimates in a lognormal model [35]. Secondly, the proposed approach allows for modeling of not just the mean but also the standard deviation/variance of the (log) BC and PM_2.5_ mass concentrations in dependence of predictor variables. This overcomes the usual homoscedasticity assumption and can provide new insights concerning effects on the pollutant variability. In addition, we utilize Gaussian processes to account for unexplained spatiotemporal correlations in the observed measurements. This is similar to, e.g., Pirani et al. [36], Kriging or linear mixed models [38]. The distributional regression approach is implemented in the Bayesian model framework provided in the R package *bamlss*, which was extended to allow for logNNC models and offers uncertainties for the model estimates. In this investigation, we aim to better understand personal exposure in urban areas using a high spatial and temporal resolution dataset from MM. Specifically, we want to quantify the impacts of meteorology, spatial and temporal factors, and traffic on the ambient BC and PM_2.5_ mass concentrations in urban areas with which pedestrians may be exposed to. To achieve this, we developed a flexible statistical model suitable for addressing the challenges arising from MM datasets, and apply it on MM datasets from two European cities: Leipzig, Germany and Rome, Italy.

## Methods

### Mobile measurement datasets

MM were taken at two locations. In 2016, year-long MM were done in Leipzig [10] (~600,000 inhabitants) which is within a low emission zone (area where gasoline vehicles with Euro 1 or higher and diesel vehicles with Euro 3 (with diesel particle filter) or higher are allowed to enter) since 2011. The 5.5-kilometer (km) MM route east of the city center included different microenvironments. MM or “rounds” were done twice per day, one between 5:00 and 11:00 a.m. and one between 14:00 and 17:30 p.m. The local rush hours occur at 7:00 a.m. and at 15:00 p.m. to 16:00 p.m. (tomtom.com, accessed on 11.03.2021). During the winter (18/01–04/03), 55 rounds were made (53 on weekdays, 2 on weekends) and 70 (60 on weekdays and 10 on weekends) during the summer (01/06–04/09) with a total 39,811 data points for BC and 40,329 for PM_2.5_ mass concentrations. No measurements were done during public holidays.

In Rome (4.5 million inhabitants), MM were done from 01/02 to 28/02, 2017. The 9-km route through different microenvironments included touristic areas mostly inaccessible to vehicles and under a low emission zone (entry for electric public buses and official vehicles only). The rounds were done three times a day, in the morning (8:00–10:30 a.m.), noon (13:00–15:30 p.m.), and evening (18:00–20:00 p.m.). The local rush hours typically occur from 7:00 to 10:00 a.m., peaking at 8:00 a.m. and from 16:00 to 20:00 p.m., peaking at 18:00 p.m. The route included a 30-min stop by the fixed station for intercomparisons with reference instruments (quality assurance is outlined in [17, 39]) located in a gated garden ~150 meters (m) away from any road publicly accessible to vehicles. Seventy-seven rounds were analyzed in this study (54 on weekdays, 23 on weekends). There were no holidays in the month of February. In total, 54,963 data points were available for BC and 53,937 for PM_2.5_ mass concentrations.

For both cities, MM were done on foot (average speed of 1.25 m s^−1^) with an instrumented backpack. The backpack is equipped with a microAethalometer (microAeth^®^, Model AE51, AethLabs, San Francisco, CA, USA) for measuring equivalent black carbon (eBC) [40], a TSI optical particle size spectrometer (OPSS, Model 3330, TSI Inc., Shoreview, MN, USA), and a GPS, all with time resolutions of 1, 10, 1 s, respectively. Details of the aerosol backpack are in the Supplementary material. PM_2.5_ mass concentrations were derived from the particle number size distributions obtained from the OPSS corrected for refractive index and the fine mode was corrected against the mobility particle size spectrometer (MPSS, size range: 0.001–0.800 µm). Details of this procedure can be found in [17]. Table S1 summarizes the description of the two measurement campaigns along with references for more details on each. The quality assurance protocols for MM using this aerosol backpack are in [17, 41] and briefly described in the Supplementary material.

### Statistical model

#### Predictor variables

The variables considered for this analysis and their respective sources are listed in Table S2. Due to the differences of the set-up of the Leipzig and Rome campaigns, the variables are not identical. The common variables are wind speed (ws) and direction (wd), day of week (weekend and weekdays, weekday), time of day (*h*), street classification (strclass), street configuration (strconf), street activities (stract), and traffic (traff). The wind speed and direction information were taken from fixed stations at urban background areas. In Leipzig, this station is ~8 km away from the route. In Rome, an air quality station within the route was where the wind information was taken and the presence of high-time resolution reference instruments allowed for the consideration of the ambient levels (amb) of the target pollutants. This was not possible for the Leipzig measurements, as there were no high-resolution data (at least 1 min) of PM_2.5_ mass concentration available. The street classification was based on official road types from OpenStreetMap [42]: “primary” streets are the highest-level streets and often part of national roads, “secondary” streets are major streets linking towns, “tertiary” streets provide access to suburbs, “residential” streets serve as an access to housing without the function of connecting settlements, and “park” are paths exclusive for pedestrians. The street configuration was based on an arbitrary assessment by the first author through onsite visual inspection of the road segments of the route: streets without buildings on both sides were considered “open”, streets with building on one side were considered as “half-open”, and streets with buildings on both sides were considered as “street canyon”. The street canyon was further split into street canyon traffic and street canyon residential where necessary. The year-long measurements in Leipzig allowed us to explore the seasonal dependence. However, “street activity” was excluded for Leipzig because there are not enough predictor variable combinations with street activities along the Leipzig route in contrast to Rome where there are more than one “street activity” possible for a particular street classification. This was done to avoid highly correlated variables (i.e., all primary streets have commercial activities, all residential streets, have only residential activities). Traffic counts were available for the main streets of the Leipzig route, which were typical/mean levels for each hour of the day for the year 2014 obtained through automatic counters by the city of Leipzig. For the Rome dataset, there exists no traffic counts on the MM route. Instead, the information on typical traffic flow was manually obtained from the traffic layer in Google Maps for each hour of each day of the week for every street that is part of the measurement route. The traffic flow is presented qualitatively in colors from green (fast), orange, red, and dark red (slow).

#### Data structure

**Fig. 1:**
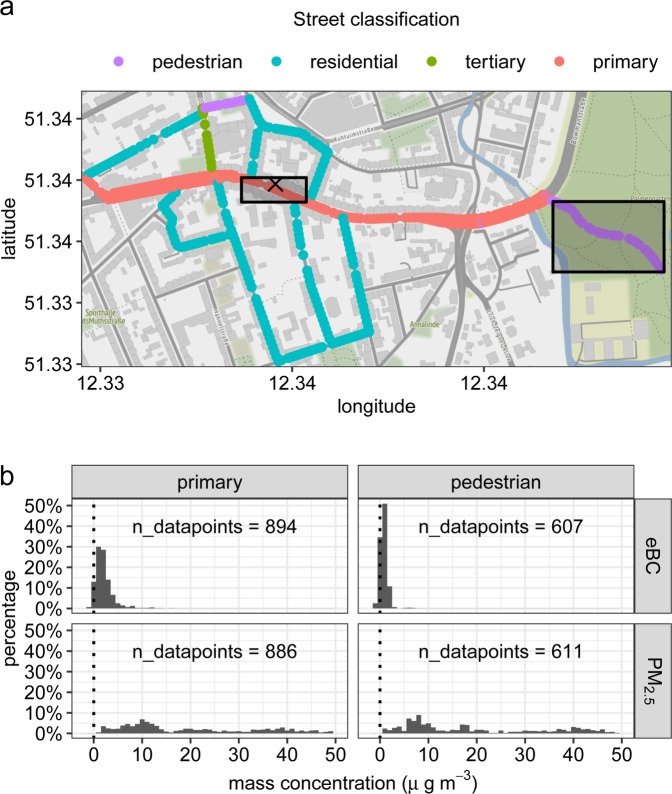
Exemplary distribution of pollutant concentrations along the measurement route in Leipzig. Measurement route in Leipzig (**a**) with centerpoints colored according to street classification. The location of a fixed monitoring station is indicated by “x”. Note that for Leipzig, no data from this station was used in this analysis. In **b**, the distributions of mass concentrations of eBC (top panels) and PM_2.5_ (bottom panels) at street classification “primary” and “park” of the MM route (in a shaded gray box in the map (**a**)) from MM done in Leipzig during winter. “n_datapoints” indicate the number of data points for each distribution. The map source is OpenStreetMap^®^ plotted with “ggmap” package in R.

Figures 1 and S1 present the distributions of eBC and PM_2.5_ mass concentrations (in micrograms per cubic meter: µg m^−3^) at a pedestrian-only street and a primary street for both Leipzig and Rome, respectively, illustrating the skewness of the data. Additionally, eBC measurements have negative values, which is normal for filter-based measurements of light attenuation with high-time resolution, where the values are a result of the difference between two consecutive readings. These negative values are part of the internal noise level of the eBC instrument. For locations with low concentrations as in Leipzig, this is even more prevalent. As this is a sampling noise that averages to zero (Fig. S2), removing the negative values risks overestimating the eBC mass concentrations. This measurement uncertainty must be accounted for to minimize the uncertainties of the model estimates as suggested by previous studies listed above [33, 36, 37]. The dataset of the eBC mass concentration is an aggregation of the raw data (1-s) to 10-s median, which still has some recognizable influence from the measurement noise. This is not applicable to the dataset of the PM_2.5_ mass concentrations, because these values were obtained after a multi-step, post-process calculation resulting in only positive values.

The Leipzig and Rome routes took 1 and 2.5 h to cover, respectively. Naturally, spatial and temporal correlations could occur and would have to be considered to achieve valid inference.

Finally, the uncertainties for the impacts that these predictor variables have on the pollutant concentrations must be calculated. Therefore, the statistical model must satisfy the following criteria:

- Account for measurement error of the eBC mass concentrations,
- account for the spatial and temporal correlations; and
- provide uncertainties for the estimated impacts of the predictor variables.

### Model description: lognormal distributional regression

To address the skewness of the datasets, we model the pollutant concentrations *Y* using a lognormal model [32–34, 36, 43]:

1 $$Z = \log Y \sim N\left( {\mu ,\,\sigma ^2} \right)$$

with mean *μ* and variance *σ*^2^ which depend on a set of predictor variables *x* and, in spatiotemporal models, on locations *s* and time *t*. We employ a Bayesian distributional regression framework [44] to model both the mean:

2 $$\mu \left( {{{{{{{{\mathbf{x}}}}}}}},s,\,t} \right) = \eta _\mu \left( {{{{{{{\mathbf{x}}}}}}}} \right) + \gamma _\mu ^{{{{{\rm{{space}}}}}}}\left( s \right) + \gamma _\mu ^{{{{{\rm{{time}}}}}}}\left( t \right)$$

and the log-standard deviation:

3 $$\log \,\sigma \left( {{{{{{{{\mathbf{x}}}}}}}},s,t} \right) = \eta _\sigma \left( {{{{{{{\mathbf{x}}}}}}}} \right) + \gamma _\sigma ^{{{{{\rm{{space}}}}}}}\left( s \right) + \gamma _\sigma ^{{{{{\rm{{time}}}}}}}\left( t \right)$$

via an additive mean predictor $\eta _\mu \left( {{{{{{{\mathbf{x}}}}}}}} \right)$ as known from GAMs (e.g., [38, 45]) and with another additive predictor$\eta _\sigma \left( {{{{{{{\mathbf{x}}}}}}}} \right)$ for the standard deviation *σ*. Note that this model is a direct extension of standard linear regression corresponding to $\mu \left( {{{{{{{{\mathbf{x}}}}}}}},\,s,\,t} \right) = \eta _\mu \left( {{{{{{{\mathbf{x}}}}}}}} \right)$ a linear predictor and $\log \sigma \left( {{{{{{{{\mathbf{x}}}}}}}},s,t} \right) = \eta _\sigma$ a constant. The spatiotemporal structure of the data is accounted for by zero mean latent error processes comparable to random intercepts in linear mixed models (e.g., [38]): for both *μ* and *σ*, Gauss–Markov random fields (e.g [46]) $\gamma ^{{{{{\rm{{space}}}}}}}\left( s \right)$ capture spatial correlations along the routes and Gaussian processes $\gamma ^{{{{{\rm{{time}}}}}}}\left( t \right)$ with exponential correlation functions reflect temporal correlations over the campaign period. Further details are given in the Supplementary material (Section II). Conditional on these latent processes, we assume the measurements to be uncorrelated. They can represent possible factors that are not accounted for by the given predictor variables which may contribute to the error term of the regression analysis (e.g., building heights, other meteorological variables, types and fuel used by the vehicles, etc.).

For Rome, we specify a model predictor (for both *μ* and *σ*):

4 $$\eta _ \ast ^{{{{{\rm{{Rome}}}}}}}\left( {{{{{{{\mathbf{X}}}}}}}} \right) =	 \, \alpha _0 + \alpha _1x_{{{{{\rm{{ws}}}}}}} + \alpha _2\log x_{{{{{\rm{{amb}}}}}}} + \beta _{{{{{\rm{{wd}}}}}}} + \beta _{{{{{\rm{{strclass}}}}}}} + \beta _{{{{{\rm{{strconf}}}}}}}\\ \,	+ \beta _{{{{{\rm{{stract}}}}}}} + \beta _{{{{{\rm{{traff}}}}}}} + \beta _{{{{{\rm{{weekday}}}}}}} + f_1\left( {x_h} \right)$$

with intercept $\alpha _0$, linear effects of wind speed $x_{{{{{\rm{{ws}}}}}}}$ and the log-ambient concentration $\log x_{{{{{\rm{{amb}}}}}}}$ with coefficients $\alpha _1$ and $\alpha _2$, respectively, and reference encoded categorical effects $\beta$ for the remaining predictor variables. The time of day is modeled by a cubic regression spline $f_1\left( {x_h} \right)$ of the hour of the day. For Leipzig, the predictor writes:

5 $$\eta _ \ast ^{{{{{\rm{{Leipzig}}}}}}}\left( {{{{{{{\mathbf{X}}}}}}}} \right) =	 \,\alpha _0 + \alpha _1x_{{{{{\rm{{ws}}}}}}} + \beta _{{{{{\rm{{wd}}}}}}} + \beta _{{{{{\rm{{strclass}}}}}}} + \beta _{{{{{\rm{{strconf}}}}}}} + \beta _{{{{{\rm{{weekday}}}}}}}\\ \,	+ \beta _{{{{{\rm{{season}}}}}}} + f_1\left( {x_h} \right) + f_2\left( {x_{{{{{\rm{count}}}}}}} \right).$$

Here, the effect of season is expressed in $\beta _{{{{{\rm{{season}}}}}}}$. Instead of categorical traffic information, we include another cubic regression spline $f_2\left( {x_{{{{{\rm{count}}}}}}} \right)$ for the traffic counts available at the primary road. $f_2\left( {x_{{{{{\rm{count}}}}}}} \right)$ is constrained to have zero mean on primary roads and is fixed to zero elsewhere to ensure interpretability of street class effects. As the models involve comparably many free parameters, we do not include any predictor variable interaction terms, to achieve stable estimates and concise interpretability. Moreover, in sensitivity checks including street class-wise time of day effects, they were estimated generally very similar over all street classes for all models.

#### Model extension: incorporating measurement error

For the eBC mass concentration, instrument noise presented a serious issue for low mass concentrations (<4 µg m^−3^, which is the expected noise level at 1-s time resolution and flow of 50 ml min^−^^1^ according to the user manual). Instead of *Y*, we, in fact, observe $\tilde Y = Y + \varepsilon$ contaminated with some measurement error $\varepsilon$, which might be reasonably assumed independent Gaussian noise $\varepsilon \sim N\left( {0,\lambda ^2} \right)$. Naïve approaches analyze such pathologic data nonetheless with a lognormal model after, e.g., removing negatives [43]. However, ignoring the measurement error in the model and omitting negative values lead to biased results (Fig. S3). Hence, we explicitly model:

6 $$\tilde Y \sim {{{{{\rm{logNNC}}}}}}\left( {\mu ,\sigma ^2,\lambda ^2} \right)$$

with a logNNC model incorporating $\varepsilon$ into the lognormal model described for $Z = \log Y$ above. The logNNC distribution was first discussed by [35] in 1991 and later used by [47], in a Kriging context. We now model the measurement error standard deviation $\lambda$ as:

7 $$\log \lambda \left( t \right) = \eta _\lambda + \gamma _\lambda ^{{{{{\rm{{time}}}}}}}\left( t \right)$$

on log scale and without including any predictor variables as the instrument behavior should not depend on these. Besides an intercept $\eta _\lambda$, we include a temporal latent error process $\gamma _\lambda ^{{{{{\rm{{time}}}}}}}\left( t \right)$ defined as above, as we found some change in error variances over different control experiments (deterioration of internal pump with use). With this logNNC model, we implicitly assume the same model structure for the underlying eBC concentrations as we do with the lognormal model for PM_2.5_, which is assumed as error-free. Results for both models are, thus, comparable.

#### Bayesian modeling

We fit all models using the flexible Bayesian distributional regression framework by Umlauf et al., which is implemented in the add-on package bamlss [44] for the statistical programming software R [48] and extended with the logNNC distribution family. To stay close to usual non-Bayesian modeling, we chose priors to form a Bayesian pendant to penalized GAMs: for the coefficients of any linear model effects, we utilize an improper flat prior resembling a frequentist modeling approach; for spline coefficients, we specify Gaussian priors resembling quadratic penalty terms. A Markov chain Monte Carlo procedure is used to obtain sampling-based estimates and credibility intervals (CIs), the Bayesian analog to confidence intervals. The logNNC density itself involves an intractable integral [35] which we approximate using Gaussian quadrature. Further modeling details are in the Supplementary material (Section III, Table S3, and Fig. S4).

## Results

### Model evaluation and comparison: logNNC

To assess the proposed logNNC model, we compare it (Final Model: lognormal, measurement error, and *σ*^2^ modeled) against alternatives, each corresponding to the Final Model in all but one key aspect distinguishing it from common approaches in literature. Replacing the logNNC by a Gaussian distribution, the distribution assumption in Model 3 (normal, measurement error implicit, *σ*^2^ modeled) corresponds to that of a linear model directly applied to the eBC mass concentration measurements [19, 30] ignoring high skewness of its distribution. In Model 2 (lognormal, measurement error ignored, *σ*^2^ modeled), we assume a lognormal [20, 32–34] ignoring additive measurement error and omitting negatives. In alternative Model 1 (lognormal, measurement error modeled, *σ*^2^ constant), the usual constant variance *σ*^2^ assumption is made. Note that depending on pollutant, measurement device, concentration level, post-processing, available predictor variables and other factors, the role of these aspects might vary. We, thus, do not want to claim that comparison results necessarily carry over to the cited analyses. They merely ought to substantiate our experience that presented alternatives reflect popular approaches in similar scenarios and, thereby, underpin practical relevance of the issue.

**Fig. 2:**
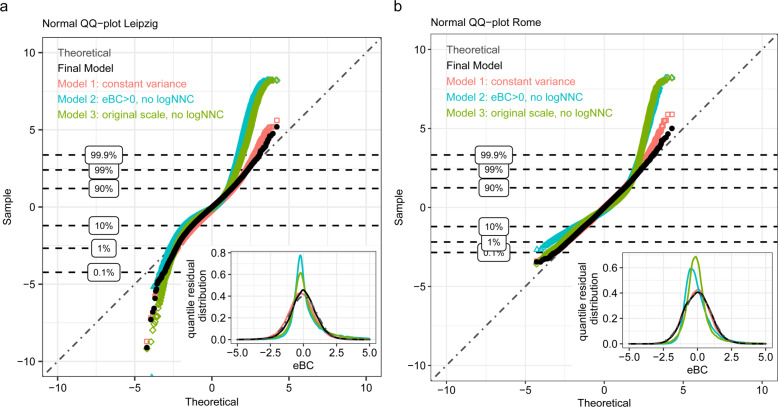
Model comparison with normal Q-Q plots of the quantile residuals. Comparison of the normal Q–Q plots of the quantile residuals of each experimental model against our model for **a** Leipzig, and **b** Rome. The inset plots show the same residual distributions in a density plot. The horizontal lines represent the quantiles of the residuals of our model. The residuals are plotted for all *n* = 39,811 data points in Leipzig and all *n* = 54,963 for Rome. Only for Model 2, the negative values are omitted. Their quantile residuals would be −∞.

**Fig. 3:**
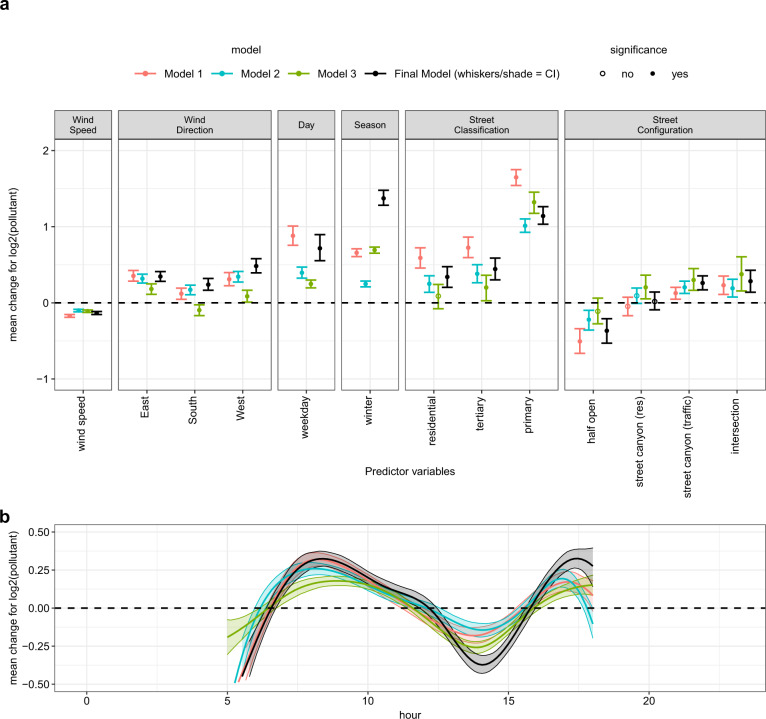
Model comparison of covariate effects. Comparison of the covariate effects on the mean *μ* of log eBC estimated in our model (Final Model) against Model 1 (constant variance, with logNNC), Model 2 (eBC > 0, lognormal), and Model 3 (original scale, no logNNC) for the predictor variables (**a**) and hour effect (**b**) in Leipzig. As in other plots in the paper, effects are marked significant when 95% CIs do not include zero, indicating that—with-in the respective model—differences from the reference group are justified. At the same time, CIs can also be used to view effect differences across models in context of internal model uncertainties. Note, that in Model 3 coefficients are not on log scale and, therefore, not quantitatively comparable with the other models. Further, mean eBC concentrations *Y* on original scale are influenced both by *μ* and the standard dev. *σ* on log scale via *E*(*Y*) = exp^f()^〖(*μ* + *σ*^2/2)〗. Thus, in terms of the mean on original scale, for instance, the positive *σ*-effect of primary streets (Fig. S10) adds to the primary street *μ*-effect depicted here—which is not the case in Model 1 with constant *σ*.

For model diagnostics, we investigate quantile residuals (Defined as $r = {{{{{{{\mathrm{{\Phi}}}}}}} }}^{ - 1}\left( {F\left( {Y|{{{{{{{\boldsymbol{x}}}}}}}}} \right)} \right)$ where *F* is the modeled cumulative distribution function of *Y* given the predictor variables ${{{{{{{\boldsymbol{x}}}}}}}}$, and ${{{{{{{\mathrm{{\Phi}}}}}}} }}^{ - 1}$ is the quantile function of a standard normal distribution. According to the inverse sampling method, *r* follows a standard normal, if *Y* indeed follows *F*.) which, under a hypothetical true underlying model, would be standard normally distributed. Thus, we may apply standard residual diagnostics from linear models, such as normal quantile–quantile (Q–Q) plots: for a good performing model, the empirical quantiles of the residual distribution (*y*-axis) would approximately match the theoretical standard normal quantiles (*x*-axis) leading to points on or close to the 1:1 line. Figure 2 shows normal Q–Q plots of our eBC models for both Rome and Leipzig compared against Models 1, 2, and 3. Models with the logNNC extension showed close to normally distributed quantile residuals suggesting a good model fit, in contrast to those without (Models 2 and 3). For the Leipzig model, strong deviations are observed in both upper and lower tails of Models 2 and 3, suggesting a heavily tailed residual distribution which is also evident in the inset plots. On the other hand, the Rome model with typically higher eBC mass concentrations showed a strong deviation mostly in the upper tail for Models 2 and 3 indicating a right-skewed residual distribution. This confirms that when ignoring the specifics of the data the model fails to capture the underlying distribution. Moreover, note that, compared to a standard model, Model 3 already benefits greatly from the additional predictor for the log mass concentration variance *σ*^2^ as, being bounded by zero, the pollutant distributions tend to become more peaked toward lower concentrations. Observed discrepancies also manifested into concrete effects on the coefficient estimates (Fig. 3). By ignoring the measurement error and omitting negative values in Model 2, covariate effects were under-estimated with all but one coefficient estimate biased toward zero: low concentration levels are over-estimated and, relatively, affected more by the additive noise than higher concentrations. This masks underlying effects and corresponds to the simulated example shown in Fig. S3. For Model 1, some estimated coefficients deviate in negative and some in positive direction. Here, due to the constant variance assumption on log scale, variability on the original scale is fully attributed to *μ* instead of explaining it by both *μ* and *σ*. Qualitatively, all models including Model 3 give similar, and to the largest extent, consistent results, although the latter is not quantitatively comparable to the others (model estimates are for log(eBC) while Model 3 results are for eBC in the original scale). While the Final Model and Model 1 fit similarly well, the Final Model adapts slightly better to the most extreme 1% of the residuals toward the right side of the distribution. Apparently, by modeling *σ*^2^ in dependence of predictor variables, unusually high values which would otherwise be considered as outliers, can be explained by the model. For Leipzig, certain divergence in the lower tail of the Q–Q plot indicates slight deviations from the assumed model. The affected smallest 1% of the residuals reflect the most negative part of the eBC measurement distribution (median −2 µg m^−3^) and show neither spatial nor temporal structure. This might suggest that the true error distribution exhibits somewhat heavier tails than the assumed normal. However, considering the huge amount of data points analyzed for both models, the majority of the residual distribution (1–99th percentile) are in complete accordance with the theoretical distribution. Thus, the logNNC model distinctly improves the severe mismatch in the fitted distributions of Models 2 and 3.

### Model evaluation: PM_2.5_ models and latent errors

Figure S5 shows the normal Q–Q plots of the lognormal PM_2.5_ models. Both plots show considerable deviation on the upper tail, and the distribution of the residuals from the Rome PM_2.5_ dataset is more normally distributed compared to Leipzig. This might be attributed to the differences on how they were calculated. In Rome, the daily intercomparison against an MPSS allowed for “run”-resolved fine mode correction factors. In Leipzig, only one correction factor was used to correct the fine mode of the volume size distribution from the OPSS. Nevertheless, the 0.1–90% of the residuals fall in the theoretical line which is satisfactory for the current possibilities of calculations of PM_2.5_ mass concentrations from optical size spectrometers.

By employing the latent spatial and temporal error processes $\gamma ^{{{{{\rm{space}}}}}}(s)$ and $\gamma ^{{{{{\rm{time}}}}}}\left( t \right)$, it was possible to widely, yet not entirely, decorrelate the residuals (see Fig. S6 for the autocorrelation functions). In Leipzig and Rome, we observe a residual autocorrelation of 0.35 and 0.51 to the subsequent measurement (i.e., after 10 s) and 0.09 and 0.20 to a measurement 5 min apart, respectively. Therefore, we want to stress that the resulting effect CIs are likely slightly too narrow. However, the large size of the datasets makes it hard to account for more fine-grained correlations while keeping the good interpretability of the model.

Finally, we observe that the estimates for the measurement error standard deviation $\lambda \left( t \right)$ in eBC mass concentration models for both Leipzig and Rome attain realistic values matching results from five control experiments (Fig. S7).

### Model results

We first focus on the estimated effects of the predictor variables on the mean (*μ*) of the log pollutant concentrations offering the most accessible interpretation. Note that we term an effect “significant” whenever the CI does not include zero. Moreover, we interpret the effects ceteris paribus, i.e., when keeping all other variables including the standard deviation *σ* fixed. Then, the effect values on log scale translate to relative, percentual changes in concentrations. Subsequently, we add a distributional perspective by illustrating model results and potentials beyond mean regression. Note that the results for Rome are limited to the cold season. We suggest caution when extrapolating these to other seasons.

**Fig. 4:**
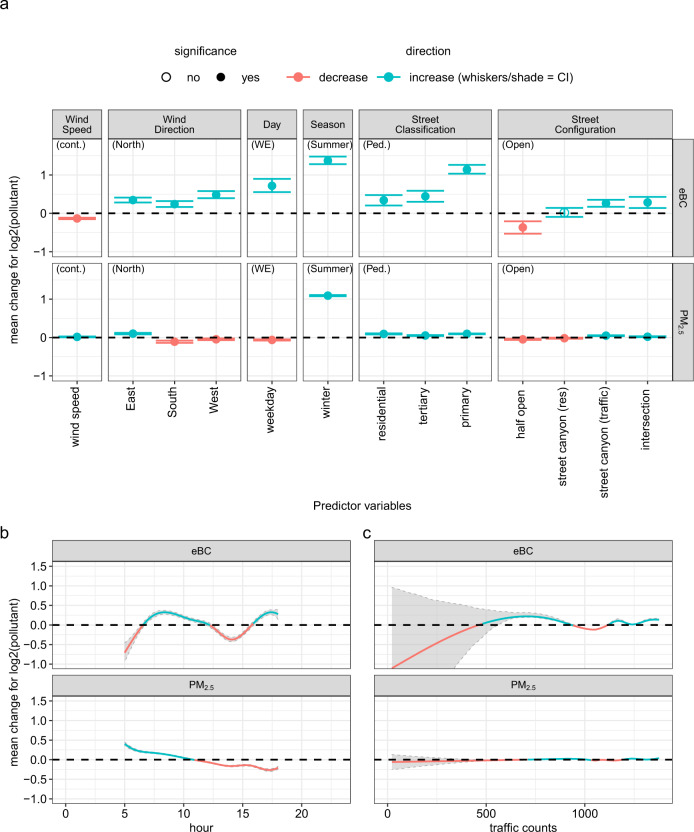
Model results of effects of covariates on μ for Leipzig. Results for the linear predictor effects (**a**), for the non-linear effect of “hour of day” (**b**) and (**c**) “traffic counts”. Effect estimates are given on log2-scale to ease interpretation (an effect of 1 doubles, −1 halves the expected pollution value ceteris paribus). For categorical predictor variables the reference category (zero effect) is given in brackets. Whiskers/shade indicates 95% credibility intervals (CI) resulting from the Bayesian model fit. If the shade is not visible, it means the CI is narrow.

**Fig. 5:**
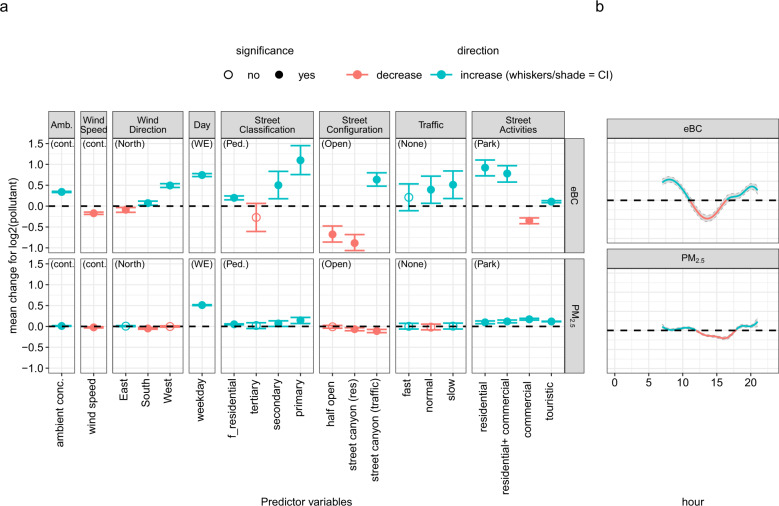
Model results of effects of covariates on μ for Rome. Results for the linear predictor effects (**a**), and the non-linear effects of “hour of day” (**b**). For further plot description see also the caption of Fig. 4.

Figures 4 and 5 show the effects of the predictor variables on the mean log eBC and PM_2.5_ mass concentrations for the Leipzig and Rome models, respectively. Here, similarities were observed in the model results from both datasets. For the eBC models, the effects of spatial characteristics such as street classification and configuration are similar in the two cities with the largest effect when changing from a park/pedestrian-only area to a primary street: in this case, the mean concentration is more than doubled with 114% or 159% increase in Rome and Leipzig, respectively. Similarly, moving from an open street to a street canyon (traffic) results in a 55% and 20% eBC mass concentration increase in Rome and Leipzig, respectively.

PM_2.5_ mass concentrations showed weak dependency on spatial characteristics for either city. PM_2.5_ mass concentration did not vary much from a pedestrian street to other settings of the street classification. The results also show insignificant effects of traffic flow on PM_2.5_ mass concentration in Rome. In urban areas, air pollution is often dominated by TRAPs and BC particles originate from these vehicular combustion processes. PM_2.5_ mass concentration, on the other hand, is a bulk mass of undifferentiated PM which could originate from particles transported over distance and has less to do with local sources. The relative independence of PM_2.5_ from local spatial features is also evident in the spatial latent errors (Figs. S8 and S9).

This is further supported by the effects on eBC mass concentration of day of week, which is closely linked to emission patterns related to human activities. For Rome, weekday concentration increases by 88% from weekends. The same can be observed for Leipzig. However, the PM_2.5_ model of Rome shows a slight effect from the temporal factors which is not the case in Leipzig. With the limited data gathered in Leipzig, the weekend-weekday variation was low and only captured changes in local sources (fewer human activities) resulting in clear effects on eBC mass concentration only.

For the effect of time of day on eBC mass concentration, we observe strong similarities between Rome and Leipzig: increase in the morning and evening, and a decrease in the middle of the day. Naturally, this can be attributed to increased human activities during rush hours. In addition, the low temperatures during the mornings and evenings result in the accumulation of particles in the urban surface layer, and high temperatures during the day promotes the mixing of polluted air below with the clean air above. Therefore, the dynamics of the vertical mixing may also influence the PM_2.5_ mass concentration. However, in contrast to eBC, the pattern of the effect on PM_2.5_ mass concentration on both cities is not entirely similar. In Rome, we observe a similar pattern to eBC mass concentration. For Leipzig, the effect of hour of day on PM_2.5_ mass concentration continues to decrease to the early hours of the evening. This could be because, unlike Rome, the measurements in Leipzig did not occur as late in the night (only up to 17:30 local time) and the evening rush hour in Leipzig occurs much earlier (from 15:00–16:00 local time). Therefore, in Leipzig, on average over seasons, the evening rush hour and the nighttime cooling of the urban surface layer do not coincide in time, resulting in different effects on eBC and PM_2.5_ mass concentrations.

The impact of traffic was also evaluated; however, the effect is much clearer in eBC mass concentration in Rome than in Leipzig. This could be attributed to traffic data used for the Leipzig model where only average traffic counts from 2014 were available.

For the case of Leipzig, a strong effect of season was observed; eBC mass concentration increases by 160% during winter compared to summer, and PM_2.5_ mass concentration increases by 119% during the wintertime. The meteorological conditions during winter promote the accumulation of particles on the urban surface layer due to the less vertical mixing of clean air from above. Hence, this affects both pollutants.

**Fig. 6:**
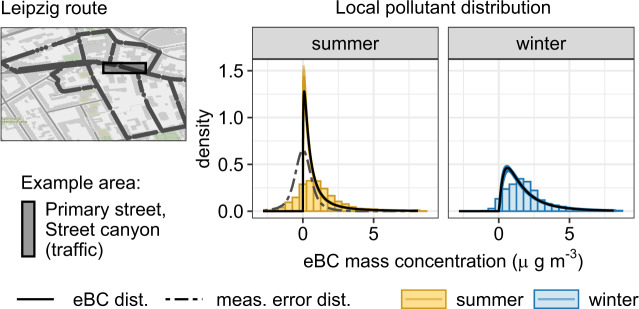
Illustration of the logNNC model results on a distributional level: for an exemplary segment of the Leipzig route, seasonal differences in the empirical eBC distribution are depicted (shaded histograms). Corresponding densities resulting from the model are deconvoluted into eBC distribution (black solid lines) and measurement error distribution (gray dashed line). Colored fading shades around model eBC densities reflect estimation uncertainties (presenting 500 posterior samples).

Aside from effects on the mean mass concentrations, a change in the pollutant concentration level typically comes with a change in variance: measurements in areas with a higher concentration level tend to have higher variability than in lower levels. In part, this is already accounted for by modeling the mean in a lognormal model. Here, an increase in the mean of the log concentration mathematically implies a certain increase in the assumed variance on the original scale. However, explicitly modeling the standard deviation (*σ*) of the log mass concentration depending on the predictor variables provides an opportunity to further adapt to the distribution of the data. Estimated effects on *σ* can be found in the Supplementary material (Figs. S10 and S11, for Leipzig and Rome, respectively). In Fig. 6, we demonstrate an example of how the predictor variable “season” affects the (modeled) distribution of the eBC mass concentrations in a central segment of the Leipzig route (street class “primary”, street configuration “street canyon (traffic)”). To achieve comparability with the empirical distributions, we aggregated the model densities, initially depending on temporally varying predictor variables, such as “wind speed”, over the seasons. We depict logNNC model densities deconvoluted into the target distribution of the local eBC mass concentrations and the distribution of the instrument measurement error (only shown on the left). The comparably large share of negative values in the summer season likely arises from many low eBC concentrations and we expect the underlying eBC density to be highly peaked close to zero. An overall increased eBC level in the winter season goes hand in hand with increased spread of the distribution of concentrations.

## Discussion

We analyzed the spatiotemporal distributions of eBC and PM_2.5_ mass concentrations in two European cities with data from MM. MM comes with some challenges for statistical methodologies. Besides their spatiotemporal correlation and right-skewed distribution, measurements might show high variability leading to a comparably low fraction of variance explained by the model, which might deem unsatisfactory at first glance. We experienced this phenomenon in our data but also, e.g., van den Bossche et al. [23] describe similar issues. When realizing, however, that in MM, concentrations are recorded across permanently changing microenvironments and subject to impacts of sudden events (e.g., smokers, turbulence) which cannot be accounted for by available predictor variables, high variability appears rather natural. This becomes particularly evident considering the extreme example of internal instrument noise in the eBC monitor. With a noise standard deviation of around 0.6 µg m^−3^, we observe median measurements of 0.44 µg m^−3^ in pedestrian areas and 1.25 µg m^−3^ in primary roads in Leipzig. Assuming, in a thought experiment, that these values present a pair of true concentrations underlying two measurements, even predictions from a theoretically perfect model would only achieve a share of around 0.33/(0.33 + 0.6^2^) ≈ 50% variance explained (corresponding to *R*^2^) (with 0.33 the variance of the two concentrations). This illustrates that, especially in MM offering numerous measurements, the fact that concentrations are stochastic itself is not a problem—it does, however, suggest a probabilistic, distributional perspective on the analysis. Similarly, while pre-aggregation of MM into segments/periods [22] can certainly present a useful measure, it also masks the original variability of the data to some degree. Consequently, obtaining a higher *R*^2^ after a higher degree of aggregation can be expected [29], which is, however, largely a mathematical consequence and does not imply that models based on more aggregated measurements would capture the original distribution of the concentrations better. With this in mind, we only applied minor aggregation in our datasets (10-s medians) and employed a distributional modeling approach. The proposed Bayesian lognormal model is based on the popular loglinear model (accounting for a skewed distribution) and extends it by several building blocks addressing different challenges arising in MM. Some of these building blocks are well-known, namely GAMs allowing for non-linear effects (e.g., [31]) and Gaussian error processes for modeling spatiotemporal correlations (e.g., [36]), while simultaneous modeling of the concentration standard deviation has, to our knowledge, not been employed before in the MM context. Finally, we proposed a logNNC model extension for eBC (accounting for the inherent instrument noise) which captures the observed distribution quite well in both study areas and distinctly better than comparable simplified approaches. It is of particular importance as the instrument used in this study to measure eBC mass concentrations is the most commonly used portable instrument for MM and exposure studies. Provided free software can be directly applied also by other researchers and flexibly adjusted to other study designs.

Our main aim is to qualitatively and quantitatively understand and compare the drivers of the spatial and temporal variabilities of eBC and PM_2.5_ mass concentrations in urban areas. To this end, we study a selected set of predictor variables which, besides general availability, contains enough variables to provide a meaningful picture while not too many to distinguish their influence in the present data situation (statistically but also conceptionally). Accordingly, we refrained from (automated) variable selection techniques, as we focus on model interpretation and statistical inference after such a selection procedure can be problematic (post selection inference) [49], and additional complexity would not add to the present investigation (even though our bamlss implementation would directly allow gradient boosting-based variable selection or Bayesian LASSO-type penalization). Our model results show the eBC mass concentration in urban areas studied here is more influenced by spatial characteristics such as street classification, configuration, and traffic than PM_2.5_ mass concentration is. This difference is also more clearly shown by the effect of time of day as well as the difference between weekdays and weekends. Our model also clearly captures the effect of season on both eBC and PM_2.5_ mass concentrations in Leipzig. Similar results were reported by Yu et al. [20]. Wu et al. [9] also reported that BC at streets with higher traffic volume correlated with traffic counts but not PM_0.5–2.5_. In addition, they also reported strong correlation between BC and working days. These results are consistent with previous studies on BC and PM_2.5_ with the latter being more influenced by regional sources than local sources [9]. For further comparisons, special care has to be taken to consider differences of the individual studies, e.g., differences in MM strategy and predictor variables, limiting immediate comparability.

The model presented here has certain limitations and strengths. Firstly, the model demonstrated sensitivity to how predictor variables were obtained. For instance, using wind information from a fixed site probably diminished the explanatory power of this variable in our analysis. The modeled effect of traffic in Leipzig did not lead to any conclusive results which could be addressed by using real-time traffic counts for when the measurements were done. The arbitrariness of the “street activities” could also play a role in the uncertainties of the model results. Nevertheless, the versatility and applicability of this model as an alternative method for analyzing MM datasets can allow users to refine and explore different predictor variables. Secondly, despite not drastically improving the current model fit, modeling the variance offers the possibility of in-depth analysis spatiotemporal effects on the pollutants’ variabilities. This can be particularly useful in problem settings where variability of the concentrations is of major interest, such as for convergence analysis in these works [12, 14, 17]. Thirdly, we have only briefly touched upon the predictive power of this model. Nonetheless, this model also has the potential for predicting pollutant concentrations with considerable measurement error in areas where in situ measurement data do not exist, which could be beneficial for personal exposure estimates [33, 37]. Further developing this approach as an air pollution model is an interesting direction for future research that is already gaining traction in air pollution exposure and epidemiological studies ([50] and reference therein).

Finally, our results provide valuable insights into the associations between local environmental factors with outdoor mass concentrations of eBC as compared to PM_2.5_. This has important implications on the assessment of the spatial variability of personal exposure of pedestrians to traffic-related air pollution in urban areas which are often based on PM_2.5_ mass concentrations. The results of this study contribute to the growing evidence of the importance of eBC as a metric, not only for monitoring the effects of mitigation efforts in improving air quality, but also for the health effects of traffic-related air pollution.

## Supplementary information

Supplementary information

## Data Availability

The “bamlss” package with the logNNC extension is available in R (version ≥ 3.5.0) [44].
