# Supplementary material for: Pedestrian exposure to black carbon and PM2.5 emissions in urban hot spots: new findings using mobile measurement techniques and flexible Bayesian regression models
Source: J Expo Sci Environ Epidemiol. 2021 Aug 28;32(4):604–14. doi: 10.1038/s41370-021-00379-5 (PMC9349038; doi:10.1038/s41370-021-00379-5)
Supplement: Supplementary file 1 — Supplementary information [file 41370_2021_379_MOESM1_ESM.docx]

**Pedestrian exposure to black carbon and PM_2.5_ emissions in urban hotspots: New findings using mobile measurement techniques** **and flexible Bayesian regression models – Supplementary Information**

Honey Dawn Alas^1^, Almond Stöcker^2,3^, Nikolaus Umlauf^4^, Oshada Senaweera^3^, Sascha Pfeifer^1^, Sonja Greven^2^, Alfred Wiedensohler^1^

^1^Leibniz Institute for Tropospheric Research (TROPOS), Leipzig, Germany

^2^Humboldt-Universität zu Berlin, Berlin, Germany

^3^Ludwig-Maximilians-Universität München (LMU), Munich, Germany

^4^Universität Innsbruck, Austria

This document contains supplementary tables, figures, and texts which detail the measurement datasets and predictor variables, model diagnostics and additional model results. Finally, the effects of the predictor variables on the standard deviation of pollutant concentrations are also presented.

1. Supplementary Tables

Table S1. Descriptions of the mobile measurement campaigns used in this study.

| Description | Leipzig | Rome |
| --- | --- | --- |
| Study period | Winter (18/01 to 04/03) and Summer (01/06 to 04/09) of 2016 | Winter (01/02 to 28/02) of 2017 |
| Length and duration of route | 5.5 km; 1 hour | 9 km; 2.5 hours |
| Presence of fixed station and intercomparison period | None | Yes, 30 minutes of each round |
| Frequency  Time of measurements | 2x a day:  Morning: between 5:00 and 11:00 AM  Afternoon: between 13:00 and 17:30 PM | 3x a day  Morning: 8:00 to 10:30 AM  Noon: 13:00 to 15:30 PM  Evening: 18:00 to 20:30 PM |
| # of rounds | Winter – 55  Summer– 70 | Winter - 77 |
| # of weekday rounds  # of weekend rounds | Winter – 53  Summer – 60  Winter – 2  Summer – 10 | 54  23 |
| References | (1) | (2, 3) |

The mobile measurements in Leipzig were done in collaboration with the Saxon State Office for Environment, Agriculture and Geology (LFULG). The mobile measurements in Rome was under an overarching campaign called Carbonaceous Aerosol in Rome and Environs (CARE-2017) in collaboration with numerous European institutions.

Table S2. Predictor variables available for Leipzig and Rome. The codes for the categories used in the model equations are italicized.

| Category | | Leipzig | Rome |
| --- | --- | --- | --- |
| Ambient concentration (continuous) - *amb*   - high-time resolution of eBC and PM_2.5_ concentration from a fixed, urban background region | | No | Yes |
| Wind speed (m/s) (continuous) – *ws*   - prevailing wind speed obtained from urban background station | | Yes | Yes |
| Wind direction (categorical) - *wd* | - North (315° – 45°) - *reference* - East (45° – 135°) - South (135° – 225°) - West (225° – 315°) | Yes | Yes |
| Season (categorical) - *season* | - summer (01/06 to 04/09) – *reference* - winter (18/01 to 04/03) | Yes | No |
| Day of week (categorical) - *weekday* | - weekend – *reference* - weekday | yes | Yes |
| Time of day (categorical) - *h* | - morning rush hour - midday - evening rush hour | Yes | Yes |
| Street classification^a^ (categorical) - *strclass* | - primary - secondary - tertiary - residential - park – *reference* | Yes | Yes |
| Street configuration^b^ (categorical) - *strconf* | - open – *reference* - half-open - street canyon residential - street canyon traffic - intersection | Yes | Yes |
| Traffic – *traff*   - *count* | (categorical-Rome^c^)   - no traffic - *reference* - fast flow - normal flow - slow flow   (continuous-Leipzig^d^)   - typical traffic counts for each hour of each day of the week. Applicable only on primary streets (horizontal streets in the middle of the Leipzig MM route) | Yes | Yes |

^a^(4)

^b^ visual inspection of author

^c^ (5); Google declines to give quantitative information on the traffic layer (6)

^d^Data obtained by Verkehrs- Und Tiefbauamt (VTA), City of Leipzig, statistically processed by Saxon State Office for Environment, Agriculture and Geology (LfULG) and finally provided to the authors.

Table S3. MCMC acceptance rates for different coefficient blocks of effects on the mean $\mu$ and the std. dev. $\sigma$ of $Z=\log Y$ and the std. dev. $\lambda$ of the measurement error in the different models.

|  |  | Linear model coefficients | | | $\boldsymbol{f}_{\boldsymbol{1}}\left( \boldsymbol{x}_{\boldsymbol{h}} \right)$ | | $\boldsymbol{f}_{\boldsymbol{2}}\left( \boldsymbol{x}_{\boldsymbol{count}} \right)$ | | $\boldsymbol{\gamma}^{\boldsymbol{space}}\left( \boldsymbol{s} \right)$ | | $\boldsymbol{\gamma}^{\boldsymbol{time}}\left( \boldsymbol{t} \right)$ | | |
| --- | --- | --- | --- | --- | --- | --- | --- | --- | --- | --- | --- | --- | --- |
| City | $Y$ | $\mu$ | $\sigma$ | $\lambda$ | $\mu$ | $\sigma$ | $\mu$ | $\sigma$ | $\mu$ | $\sigma$ | $\mu$ | $\sigma$ | $\lambda$ |
| Leipzig | eBC | 0.96 | 0.91 | 0.95 | 0.99 | 0.97 | 0.95 | 0.95 | 0.78 | 0.74 | 0.94 | 0.95 | 0.77 |
| Leipzig | PM_2.5_ | 1 | 0.90 |  | 1 | 0.96 | 1 | 0.86 | 1 | 0.61 | 1 | 0.31 |  |
| Rome | eBC | 0.98 | 0.86 | 0.92 | 0.99 | 0.98 |  |  | 0.94 | 0.62 | 0.99 | 0.96 | 0.64 |
| Rome | PM_2.5_ | 1 | 0.83 |  | 1 | **0** |  |  | 1 | 0.38 | 1 | 0.65 |  |

1. Supplementary Figures
2. Descriptive figures of the dataset


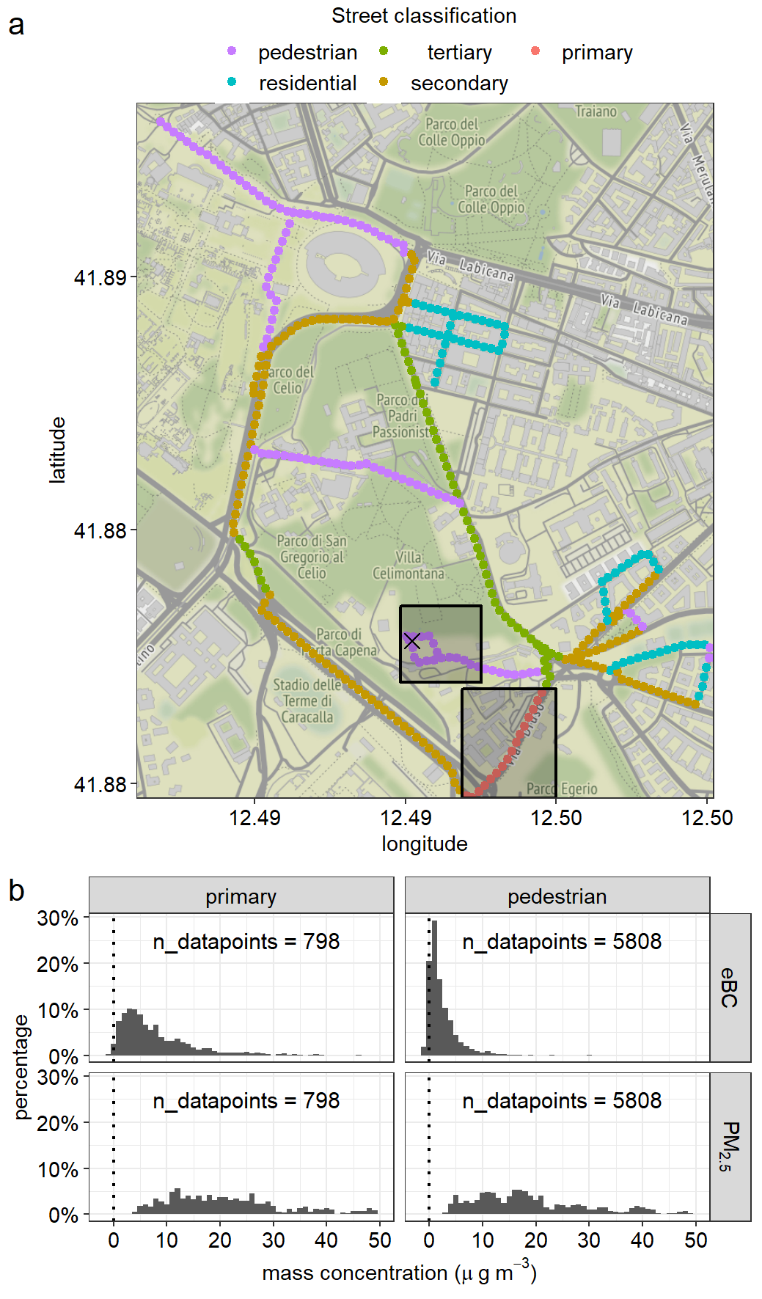


Figure S1. MM route in Rome (a) with center points colored according to street classification. The location of a fixed monitoring station is indicated by “x”. This station contained reference instruments from which quality assurance was done, and where data of ambient concentrations and wind information were measured. In the panels below (b), the distributions of mass concentrations of eBC (top panels) and PM_2.5_ (bottom panels at street classifications “primary” and “park” of the MM route (in a shaded grey box in the map (a)) from MM done in Rome. “n_datapoints” indicate the number of data points for each distribution. The map source is OpenStreetMap® plotted with ‘ggmap’ package in R.


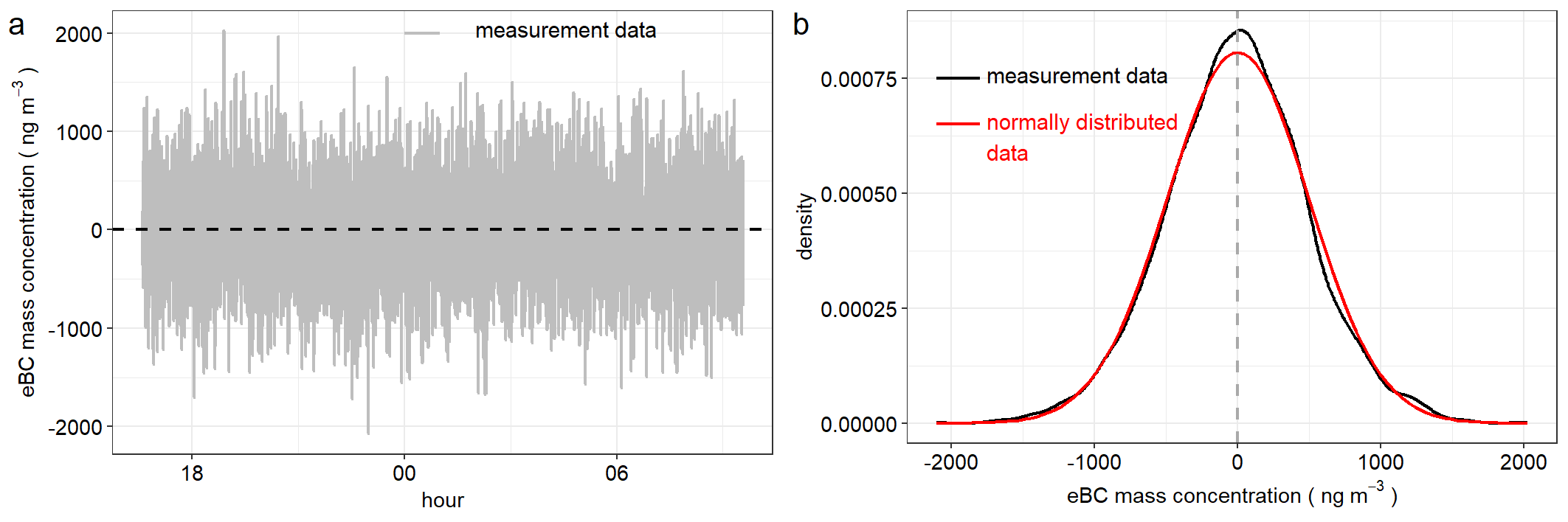


Figure S2. Instrumental noise of the AE51 operated to sample particle-free air (using HEPA filter) with flow of 150 mL/min at a time resolution of 1-s over 15 hours then aggregated to 10-s median to represent actual data used in this analysis.

- logNNC model comparison


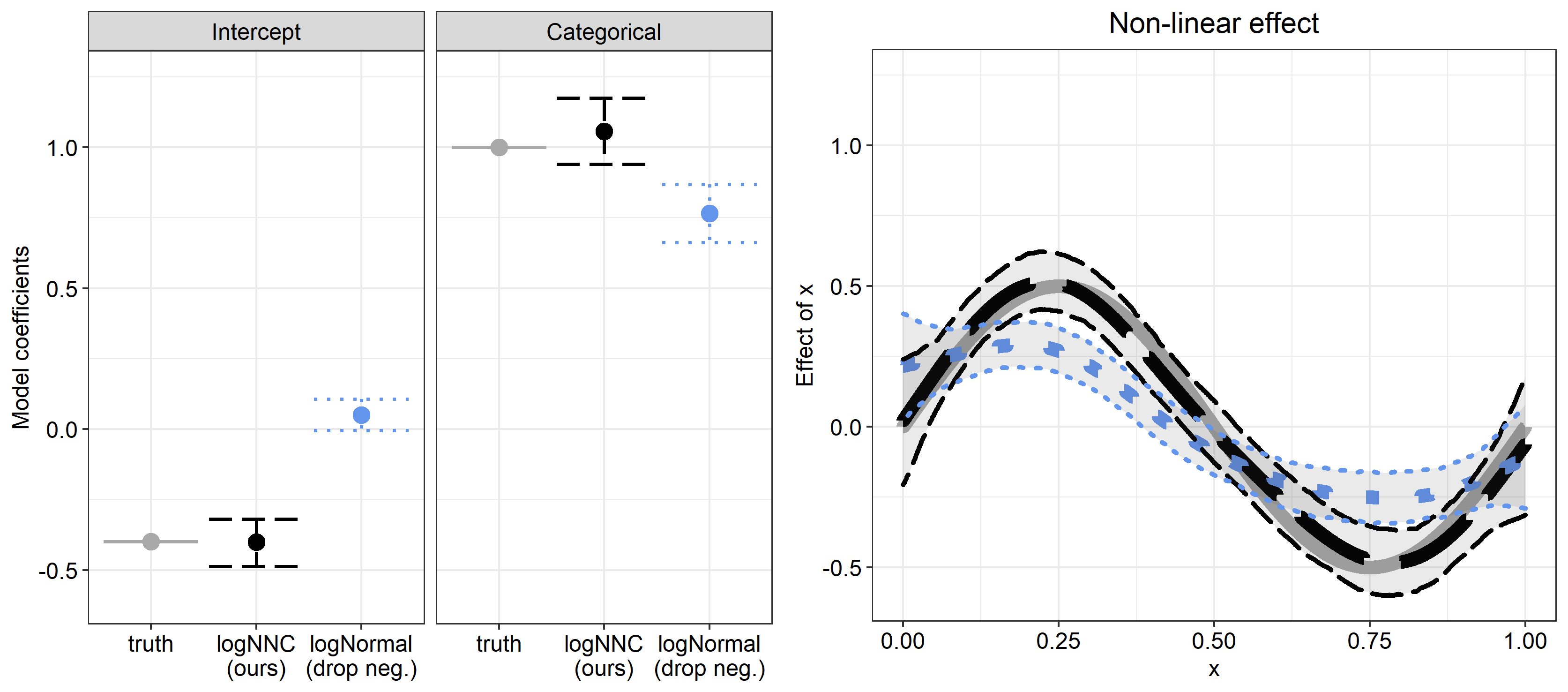


Figure S3. Estimates of a logNNC model (black, dashed lines) and a naïve lognormal model (blue, dotted lines) fitted to synthetic lognormal data with Gaussian measurement error (n=3000). True effects (grey, solid lines) include an intercept, a categorical and a non-linear effect of predictor variables on $\mu$; $\sigma^{2}$ and $\lambda^{2}$ are modelled constant; although artificial effect sizes and variances are motivated by the Leipzig eBC data. Bars / scattered lines reflect 95% credibility intervals.

1. Latent Gaussian error processes


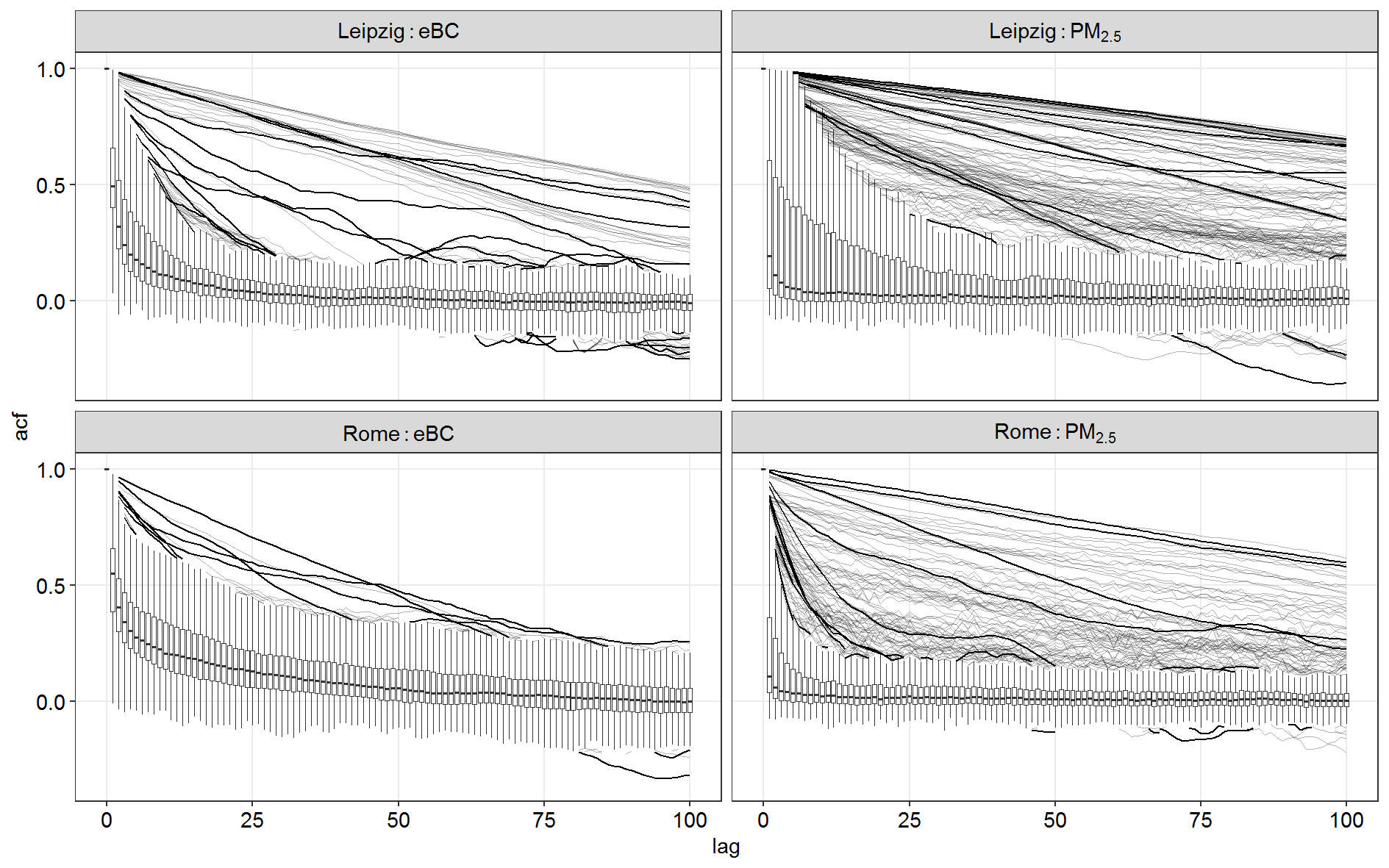


Figure S4. Lag-wise boxplots summarizing the distribution of auto-correlation functions for MCMC samples of all model parameters. Outliers belonging to the same parameter are connected by lines (linear effect coefficients highlighted in black in front of others in grey). For ‘Rome: $PM_{2.5}$’ the parameters of the daytime effect on sigma were left out, as no MCMC proposals were accepted as alternative to the posterior optimum.

1. Model behavior

- Model diagnostics
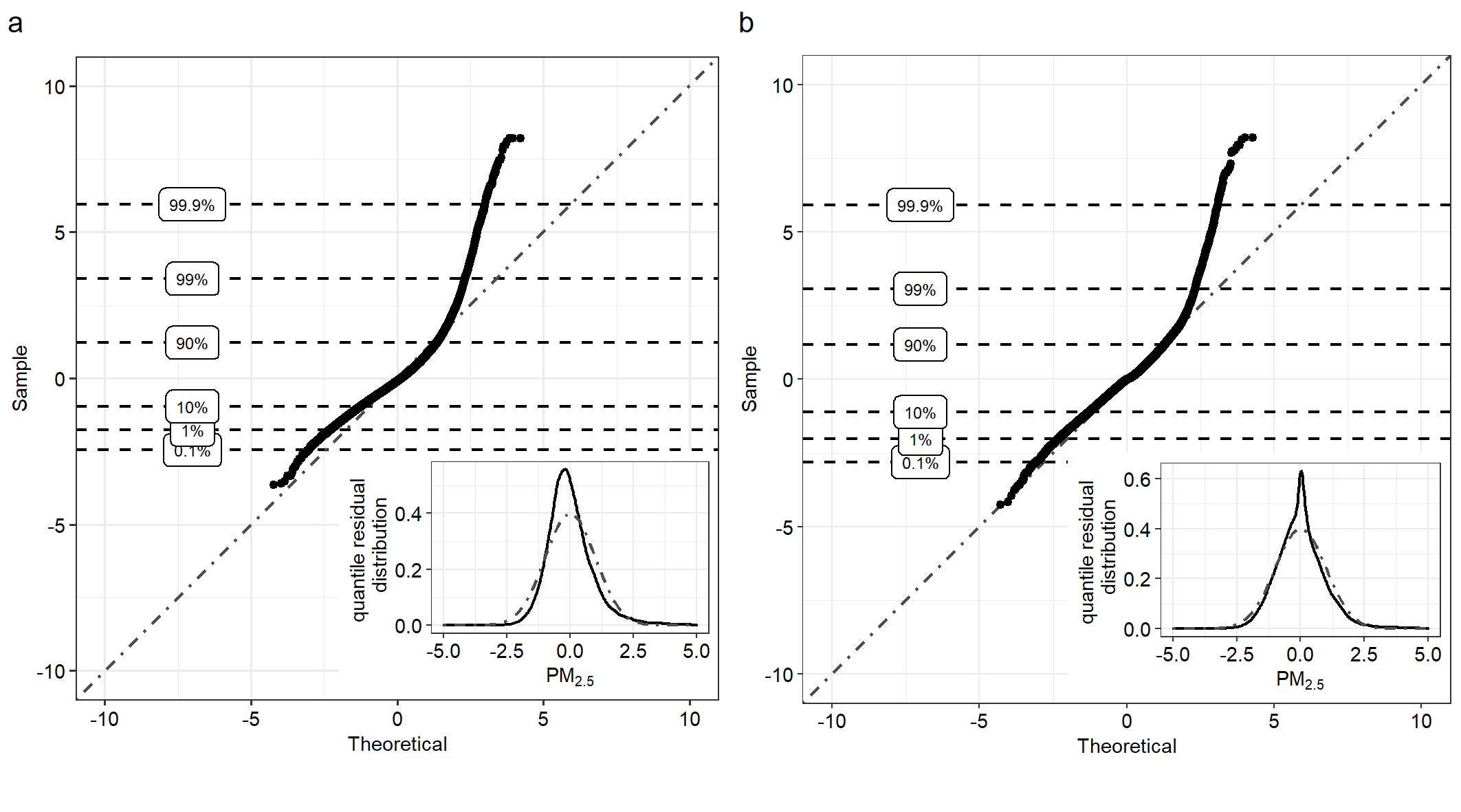


Figure S5. Normal Q-Q plots of the residuals of the PM_2.5_ models for (a) Leipzig and (b) Rome. The horizontal lines represent the quantiles of the residuals of our model. The inset plots show exactly the same information in a density plot.


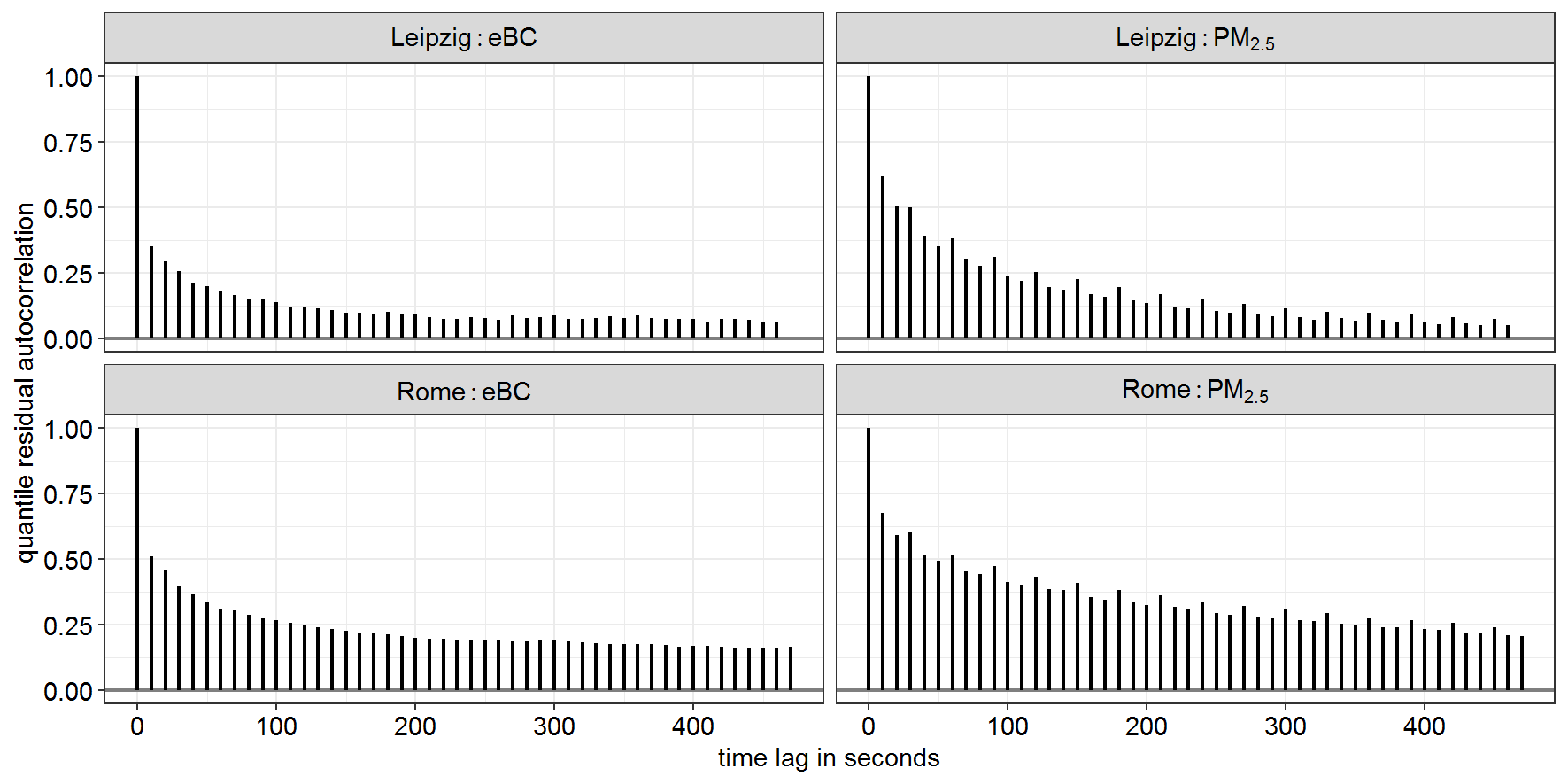


Figure S6. Empirical autocorrelation functions for the quantile residuals of the logNNC models for the $eBC$ concentration in Leipzig and Rome, and the lognormal models for the $PM_{2.5}$ concentration in Leipzig and Rome, respectively. Measurements where taken every 10 seconds.


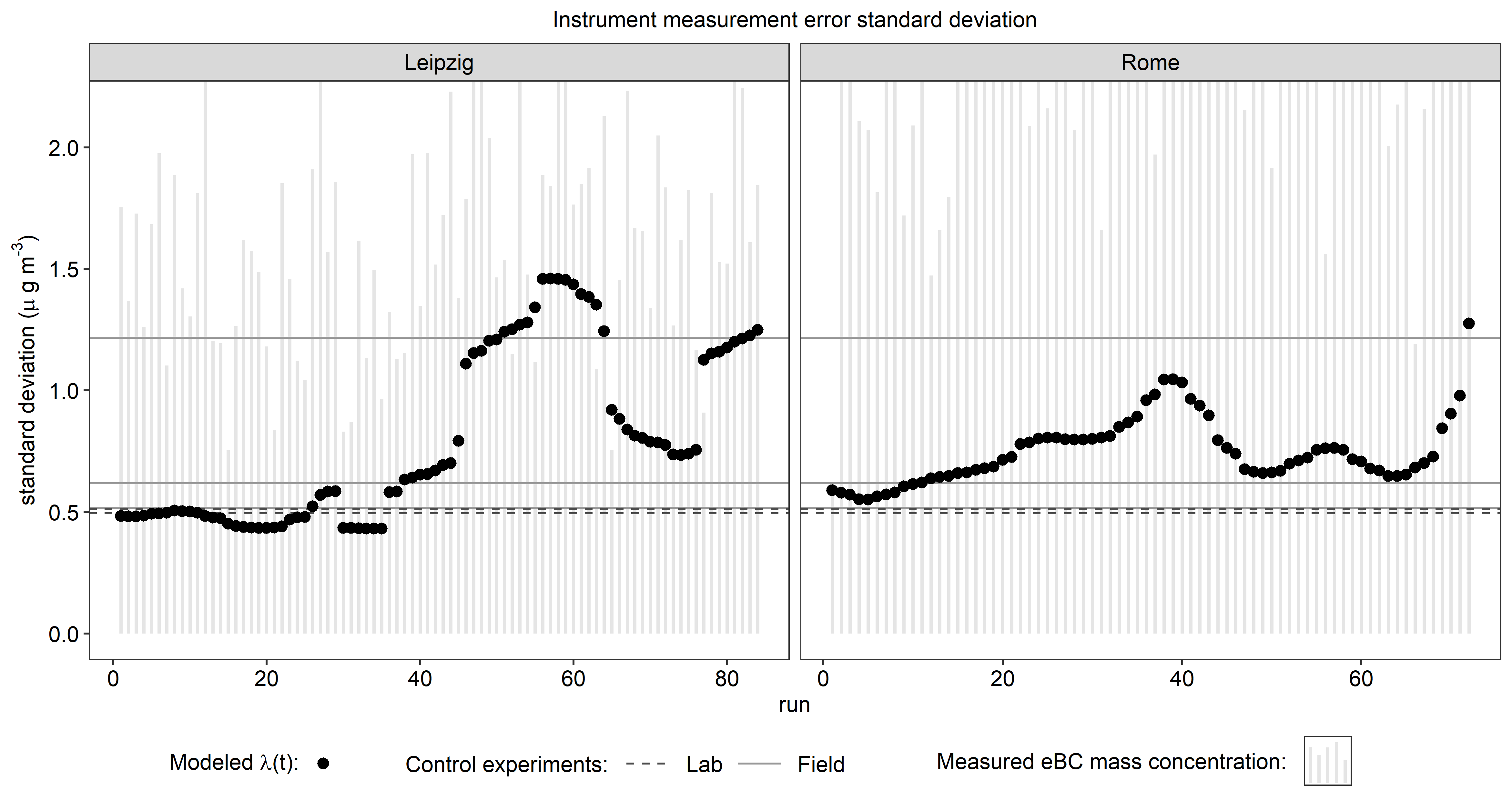


Figure S7. Model estimates of $\lambda(t)$ (measurement error standard deviation) compared against results of two laboratory and three field control experiments as shown, i.a., in Fig. S2 (dashed and solid horizontal lines, respectively). Ranging over the whole time span of the campaigns, the $\lambda(t)$ are shown averaged per run. To visualize the scale of the measurement error, horizontal grey bars are added indicating the total empirical standard deviation of the eBC mass concentration measurements observed for each run.

1. Additional model results

- Spatial latent errors on mean (µ)


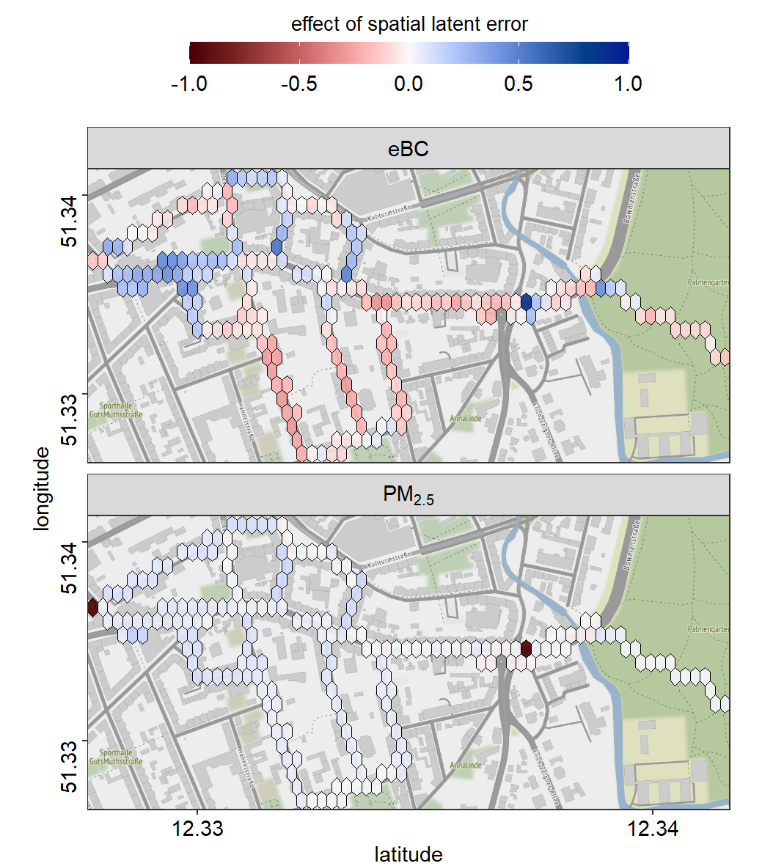


Figure S8. Spatial latent error on mean (µ) mass concentrations for Leipzig. Red colors indicate a decreasing effect and the blue colors indicate increasing effect on the pollutant concentration. The map source is OpenStreetMap® plotted with ‘ggmap’ (7) package in R.


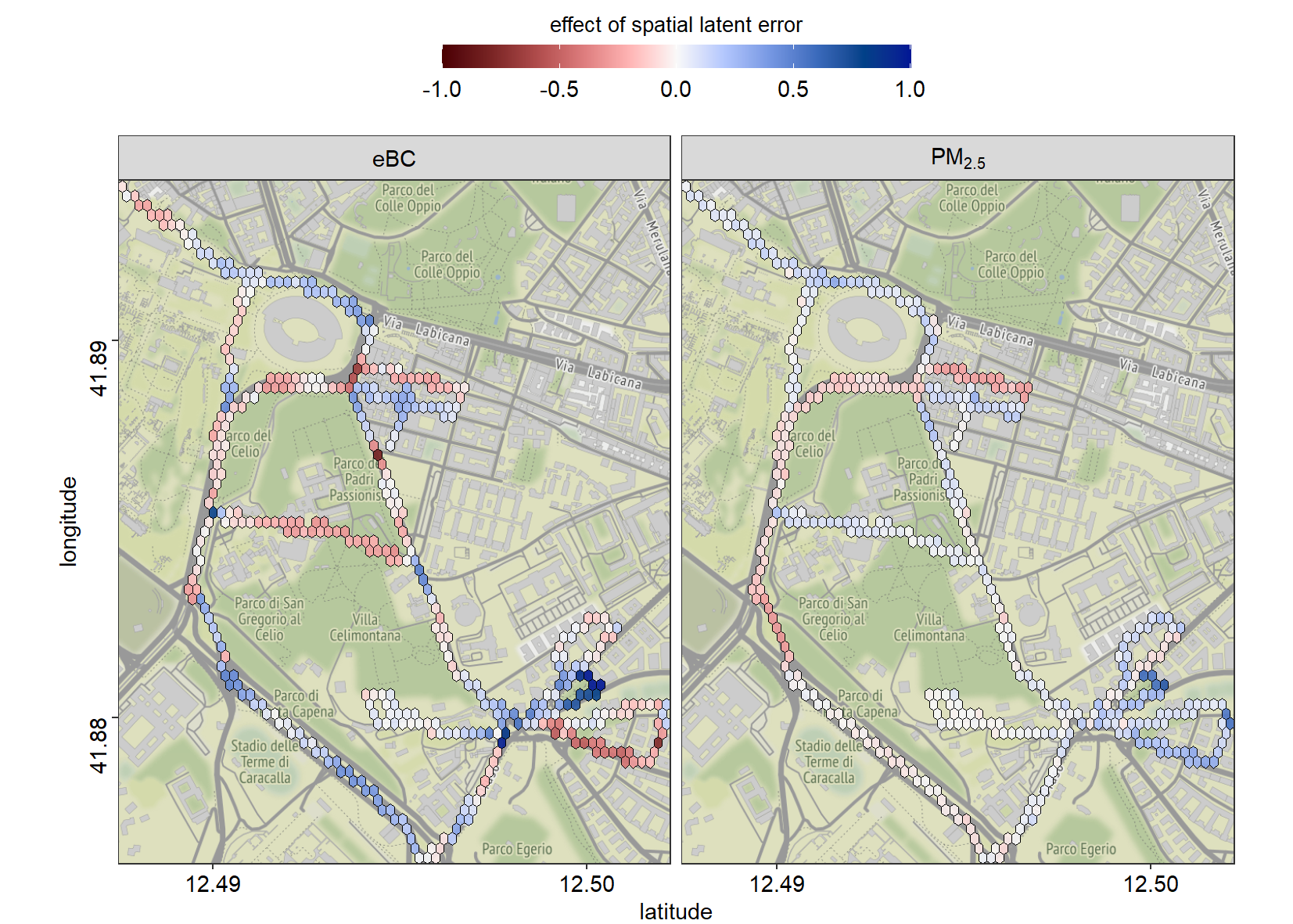


Figure S9. Spatial latent error on mean (µ) mass concentrations for Rome. Red colors indicate a decreasing effect and the blue colors indicate increasing effect on the pollutant concentration. The map source is OpenStreetMap® plotted with ‘ggmap’ (7) package in R.

- Effect of covariates on the pollutant standard deviation (σ).


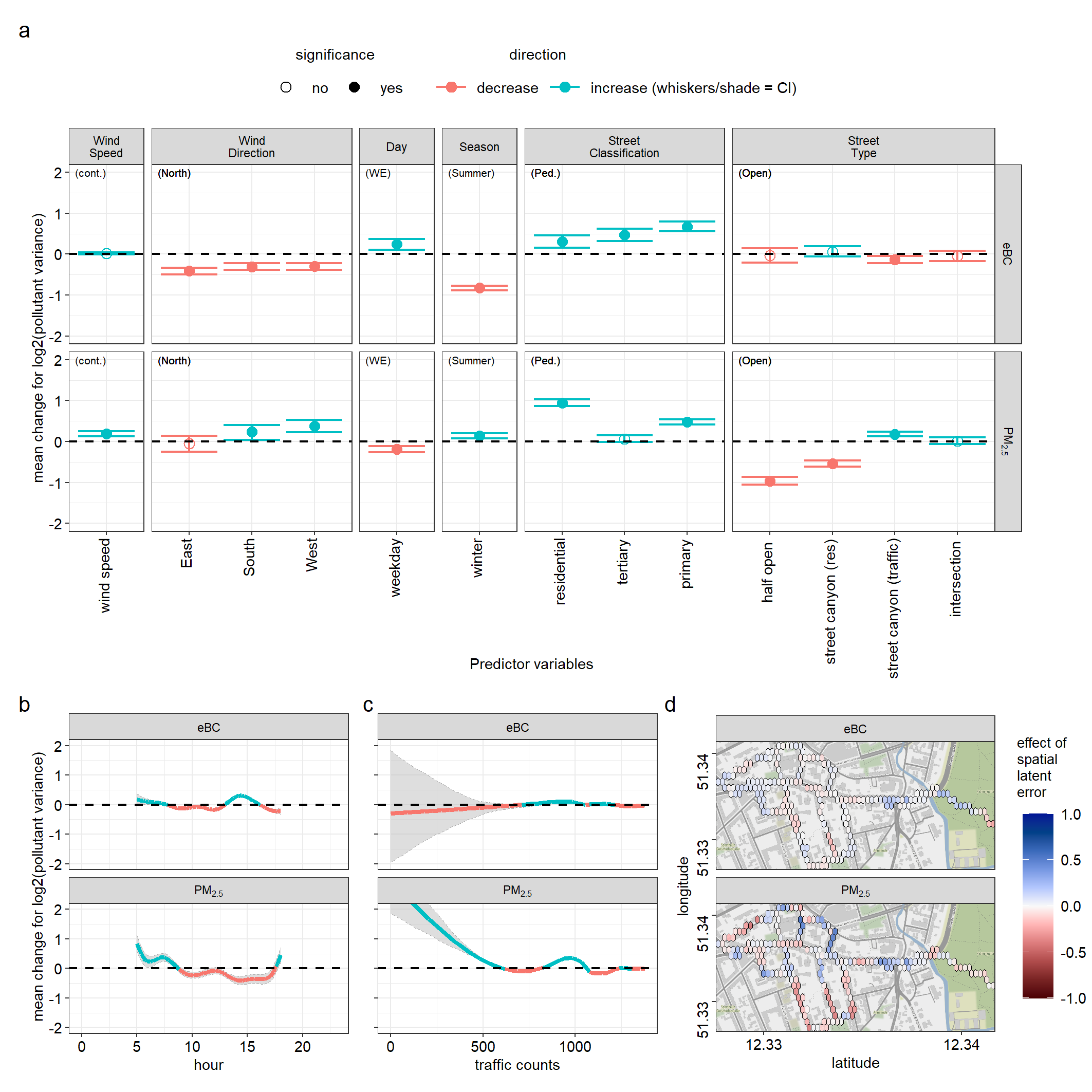


Figure S10. Effect of the predictor variables on pollutant standard deviation (log2(σ)) for Leipzig. The map source is OpenStreetMap® plotted with ‘ggmap’ (7) package in R.


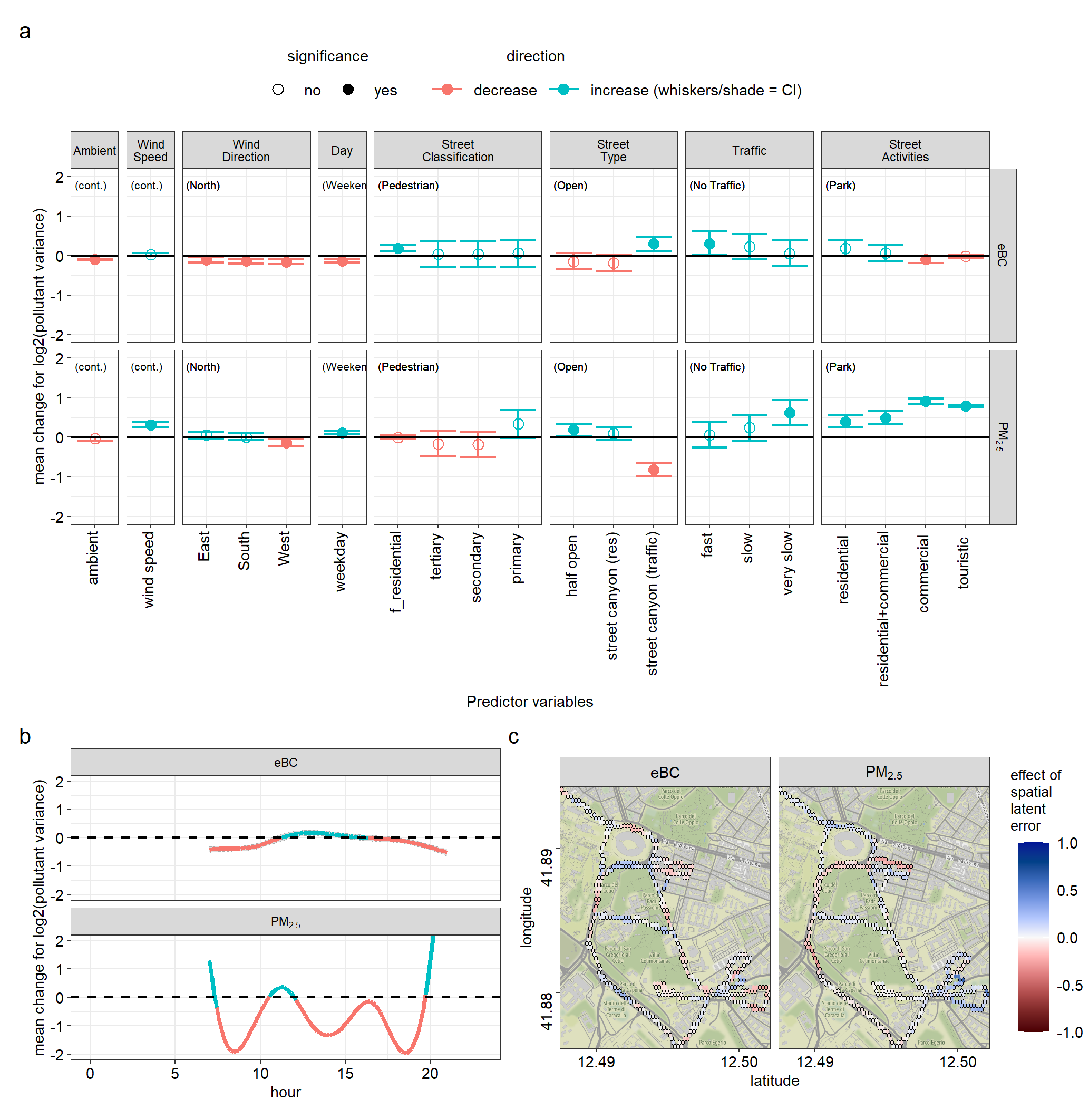


Figure S11. Effect of the predictor variables on pollutant standard deviation (log2(σ)) for Rome. For the daytime effect on (log2(σ)) in the model for $PM_{2.5}$ shown in b), no proper CI could be obtained (see Fig. S2 caption). The map source is OpenStreetMap® plotted with ‘ggmap’ (7) package in R.

1. Supplementary Information

Operation of the aerosol backpack

The aerosol backpack was developed at Leibniz Institute for Tropospheric Research (TROPOS).

A 1-m long inlet protrudes from the top of the backpack where air enters the system and is split into two streams for the two instruments. A silica gel dryer is placed prior to the inlet of the AE51 to dampen the effects of sudden changes in the environment (8). The AE51, being sensitive to vibrations (9) is secured within the backpack with a metal brace. These measures are taken to minimize instrument noise and artificial peaks in the signal. Data from the two instruments together with the GPS data are automatically logged in a microcomputer which has algorithms to control the instruments once they are manually turned on. The instruments continuously measure pollutant concentrations and the position until they and the microcomputer are manually turned off.

MM were done on foot, with volunteers/researchers walking at an average walking speed of 1.25 m s-1. The mobile measurement data is in 10-s time resolution. With continuous walking at an average speed of 1.25 m s-1, the spatial resolution is 8 m. In case of stopping due to traffic lights (crossing streets) or during intercomparison with reference instruments in fixed station, the data points are accumulated at those particular points in space.

Description of Quality Assurance of mobile and reference instruments

The quality assurance of the AE51 and the OPSS, and the entire system of the aerosol backpack is detailed in Alas et al., (3). Briefly, prior to a measurement campaign, the following quality checks are done:

- Flow checks and calibrations of each instrument and the system as a whole (also done prior to each mobile measurement round, except the flow calibration)
- Leak checks of each instrument and the system as a whole using a particle filter (also done prior to each mobile measurement round)
- Unit-to-unit variabilities (in the laboratory and in the field, in the case of the Rome campaign, this was possible during the campaign as well with another aerosol backpack with identical instrumentation)
- The instruments are compared against other units and against “reference” instruments in the World Calibration Centre for Aerosol Physics (WCCAP) at TROPOS. The AE51 is compared against a multi-angle absorption photometer (MAAP) while the OPSS is compared against a mobility particle size spectrometer (MPSS) and an aerodynamic particle size spectrometer (APSS). This is done both in laboratory settings and in the field (staying near a regular air quality monitoring station). In the Rome campaign, a fixed station with a MAAP, MPSS, and APSS is present within the mobile measurement route, providing intercomparison between the mobile and reference instruments DURING each mobile measurement round.

All reference instruments used are regularly quality-checked and calibrated at the WCCAP in TROPOS following the procedures and standards provided in (10, 11) and (12). These procedures are standard for measurements of aerosol physical properties (absorption, particle number concentration, particle number size distribution) within the framework of the pan-European research infrastructure Aerosol, Clouds and Trace Gases Research Infrastructure (ACTRIS).

The spatial latent error process is modelled as Gauss-Markov random field based on discretized grids of measurement locations $s_{1},\ldots,s_{m}$ along the routes, with $m=192$ grid points in Leipzig and $m=418$ in Rome. This means that we model $\gamma^{space}\left( s_{1} \right),\ldots,\gamma^{space}\left( s_{m} \right)$ as multivariate Gaussian with zero mean and a covariance induced by a first order Markov neighborhood structure on the route grid. Unlike in Kriging-type Gaussian processes, the correlation, thereby, reflects the distance of points along the route grid instead of the Euclidean distance on the map. We consider this more suitable in the present scenario. In contrast to the periodic daytime effect $f_{1}\left( x_{h} \right)$, the temporal latent error process $\gamma^{time}\left( t \right)$ ranges over the complete time of the campaign. For $\gamma^{time}\left( t \right)$, we apply a continuous Gaussian process with again zero mean and exponential correlation function $\rho\left( \Delta t \right)=exp\left( -a\Delta t \right)$ for a time difference $\Delta t$ and with a non-negative decay parameter $a$. Besides presenting a popular choice, we found this led to (slightly) lower residual autocorrelation than spherical and different Matérn correlation functions available in the used R package bamlss (based on mgcv^1^). Thereby, $\gamma^{space}\left( s \right)$ reflects spatially constant patterns which cannot be captured by the categorized spatial predictor variables street class and street configuration. Similarly, $\gamma^{time}\left( t \right)$ accounts for temporal dependencies not explained by the temporal predictor variables weekday and season. Before interpreting the model results, we apply suitable projections to ensure that the latent error processes do not explain any patterns that can be explained by the respective predictor variables.

Bayesian modelling details

In the Bayesian modeling approach, model coefficients/parameters are not estimated as ‘unknown but fixed’ quantities - instead the uncertainty about them is itself represented by probability distributions. Fitting the model is applying Bayes’ rule for conditional probabilities: initially specified ‘prior’ parameter distributions are updated to achieve the ‘posterior’ distribution of the parameters given the observed data. Model estimates are then given by the posterior expected parameter value (or the one with the highest probability density). The estimation uncertainty can be subsumed in credibility intervals (intervals containing the parameter with, say, 95% probability), the Bayesian analogue to confidence intervals. Practically, prior distributions can be used to regularize parameter estimation, which is particularly beneficial in complex regression scenarios like the ones presented here. The Bayesian approach yields direct valid inference also for the non-standard logNNC distribution and allows, e.g., also to incorporate estimation uncertainty naturally into the predicted distribution.

Prior parameter distributions are specified equally for all models presented and for all predictors in the models. Following the design of the R package bamlss, they are constructed as Bayesian pendant to penalized Generalized Additive Models available in the R package mgcv (compare Wood 2017^[[1]](#footnote-1)^): for the coefficients of any linear model effects, we utilize an improper flat prior resembling a frequentist modelling approach. For the basis coefficients of the cubic regression splines $f_{1}\left( x_{h} \right)$ and $f_{2}\left( x_{count} \right)$ with eight equally spaced knots (i.e. ten basis functions), a Gaussian prior is assumed with the precision matrix (the inverse covariance matrix) implementing a quadratic penalty on the second derivative of the form $\int f^{''}\left( x \right)^{2}dx$, which is a commonly used option for regression splines. For $x_{h}$, the splines are constrained to be periodic over 24h. The strength of the penalty is controlled by the variance of the coefficient prior, which presents a hyper-parameter and is equipped with another prior, an inverse-gamma prior. For the spatial and temporal latent error processes $\gamma^{space}\left( s \right)$ and $\gamma^{time}\left( t \right)$ the setup is analogous, only that here the precision matrix reflects the correlation structure of the Gauss-Markov random field and Gaussian process, respectively. For $\gamma^{time}\left( t \right)$ a complete consideration of the correlation matrix over all measurements would lead to a computationally infeasibly large number of parameters. Thus, a lower dimensional approximation is used, restricting to correlations $\rho\left( \left| t-t_{k} \right| \right)$ with a feasible number of knots $t_{k}$. The number of knots used is adapted to the dataset to ensure that enough knots are provided to achieve an acceptable reduction of the residual autocorrelation (as shown in Figure S6). For the $PM_{2.5}$ models far more knots were needed than for the eBC models. On the other hand, we had to use low-rank approximations of the spatial Gauss-Markov random field for both $PM_{2.5}$ models to achieve good MCMC mixing properties. However, we ensure that covariate effects of neither $PM_{2.5}$ models nor eBC models are at a disadvantage despite their deviations in the number of latent error process parameters: via a post-hoc projections, we constrain $\gamma^{space}\left( s \right)$ and $\gamma^{time}\left( t \right)$ to only represent structures which cannot be captured by spatial and temporal covariate effects, respectively. Again, the prior variances control the smoothness of $\gamma^{space}\left( s \right)$ and $\gamma^{time}\left( t \right)$ and are modelled via inverse-gamma priors.

For conducting MCMC simulations, the posterior mode maximizing the posterior is used as a starting value. After that, a derivative-based random-walk Metropolis-Hastings algorithm is used, where proposals are drawn from a multivariate normal distribution obtained by second-order Taylor approximation of the log-posterior centred at the current state (for details see Umlauf et al. 2018). For each model, we sample 1000 net posterior samples of 501 parameters (Leipzig: eBC), 610 parameters (Leipzig: $PM_{2.5}$), 943 parameters (Rome: eBC) and 592 parameters (Rome: $PM_{2.5}$), respectively. For the logNNC models for eBC mass concentration, all samples after a burn-in of 200 were used, while for the $PM_{2.5}$ lognormal models thinning was needed to achieve equally good mixing: here, we produced 10 times more samples and used every tenth of it. Figure S2 summarizes the MCMC sample autocorrelation. Especially given the comparably large number of parameters the results deem satisfactory: while for single parameters the autocorrelation is high, this is not the case for most of them. Satisfactory sampling is also supported by the obtained MCMC acceptance rates shown in Table S3. As all coefficients of the linear model part and of each smooth term are sampled en bloc, respectively, joint acceptance rates are reported for each group. Smoothing parameters are sampled separately with 100% acceptance, obtained from conjugate inverse Gamma distributions for single smoothing parameter model terms, or by slice sampling for multiple smoothing parameter model terms. Since, for $\mu$ in the lognormal model for PM_2.5_ mass concentrations, the utilized approximation to the full conditional posterior is exact, parameters are always accepted here. Only for the daytime effect on $\sigma$ in the Rome model for PM_2.5_, sampling fails, and proposals are never accepted. Accordingly, no CIs are obtained for this effect.

References:

1. Alas HDC, Pfeifer S, Wiesner A, Wehner B, Weinhold K, Merkel M, et al. Representativeness and variability of PM2.5 mass concentrations and black carbon near traffic and urban background monitoring stations. Gefahrstoffe - Reinhaltung der Luft. 2019;79(6).

2. Costabile F, Alas H, Aufderheide M, Avino P, Amato F, Argentini S, et al. First Results of the “Carbonaceous Aerosol in Rome and Environs (CARE)” Experiment: Beyond Current Standards for PM10. Atmosphere. 2017;8(12).

3. Alas HDC, Weinhold K, Costabile F, Di Ianni A, Müller T, Pfeifer S, et al. Methodology for High Quality Mobile Measurement with Focus on Black Carbon and Particle Mass Concentrations. Atmospheric Measurement Techniques. 2019;12:15.

4. OpenStreetMap. Highway:International equivalence: OpenStreetMap Wiki; [Available from: <https://wiki.openstreetmap.org/w/index.php?title=Highway:International_equivalence&oldid=2122789>.

5. Google. Google Maps Typical Traffic Layer: Rome [Available from: <https://www.google.com/maps/place/Rome,+Metropolitan+City+of+Rome,+Italy>.

6. GregMC. What are speed thresholds that identify traffic colors in urban areas? [Online forum comment] 2019 [Available from: <https://support.google.com/maps/thread/16003851?hl=en>.

7. Kahle D, Wickham H. ggmap: Spatial Visualization with ggplot2. The R Journal. 2013;5(1):144-61.

8. Cai J, Yan B, Kinney PL, Perzanowski MS, Jung KH, Li T, et al. Optimization approaches to ameliorate humidity and vibration related issues using the microAeth black carbon monitor for personal exposure measurement. Aerosol Sci Tech. 2013;47(11):1196-204.

9. Apte JS, Kirchstetter TW, Reich AH, Deshpande SJ, Kaushik G, Chel A, et al. Concentrations of fine, ultrafine, and black carbon particles in auto-rickshaws in New Delhi, India. Atmospheric Environment. 2011;45(26):4470-80.

10. Müller T, Henzing JS, de Leeuw G, Wiedensohler A, Alastuey A, Angelov H, et al. Characterization and intercomparison of aerosol absorption photometers: result of two intercomparison workshops. Atmos Meas Tech. 2011;4(2):245-68.

11. Pfeifer S, Müller T, Weinhold K, Zikova N, Martins dos Santos S, Marinoni A, et al. Intercomparison of 15 aerodynamic particle size spectrometers (APS 3321): uncertainties in particle sizing and number size distribution. Atmospheric Measurement Techniques. 2016;9(4):1545-51.

12. Wiedensohler A, Wiesner A, Weinhold K, Birmili W, Hermann M, Merkel M, et al. Mobility particle size spectrometers: Calibration procedures and measurement uncertainties. Aerosol Sci Tech. 2018;52(2):146-64.

1. Wood S.N. (2017) Generalized Additive Models: An Introduction with R (2nd edition). Chapman and Hall/CRC Press. [↑](#footnote-ref-1)
